# Supplementary material for: Adaptation of the Texas Christian University Organisational Readiness for Change Short Form (TCU-ORC-SF) for use in primary health facilities in South Africa
Source: BMJ Open. 2021 Dec 17;11(12):e047320. doi: 10.1136/bmjopen-2020-047320 (PMC8689126; doi:10.1136/bmjopen-2020-047320)
Supplement: Supplementary data [file bmjopen-2020-047320supp001.pdf]

Supplementary Table 1: Items removed from TCU-ORC before survey use based on research team discussion (23 total)

Item removed from original scale	Reason
A5 increasing program participation by clients	With high workloads and staff shortages this is not part of the expected role
A9 improving cognitive focus of clients during group counseling	Group counselling not currently provided
A19 improving billing/financial/accounting procedures	Clinic services provided free of charge
A30 Current pressures to make program changes come from board members or overseers	Stakeholder group not influential in government run and funded health facilities
A31 Current pressures to make program changes come from community groups	Stakeholder group not influential in government run and funded health facilities
A32 Current pressures to make program changes come from funding agencies	Stakeholder group not influential in government run and funded health facilities
A33 Current pressures to make program changes come from accreditation or licensing authorities	Stakeholder group not influential in government run and funded health facilities
B6 Policies limit use of the Internet for work-related needs at your program.	Most clinic staff do not have internet access at work or use for work purposes
B7 Computer problems are usually repaired promptly at your program	Most clinic staff do not use computers in day to day work
B10 You have convenient access to e-mail at work	Most clinic staff to not use email for work purposes
B14 More computers are needed for staff in your program to use	Clinic staff not expected to have computers for work use
B15 Most client records for your program are computerized	Electronic records system not implemented
B21 You used the Internet at work recently to access drug treatment information.	Clinic staff do not use internet for work purposes
B23 You have easy access for using the Internet at work	Clinic staff not expected to have internet access for work purposes
B26 You have a computer to use in your personal office space at work	Clinic staff not expected to have computer for work purposes
B29 The budget in your program allows staff to attend professional training.	General staff do not have knowledge about budgets and allocations, in service training most common
B30 Staff in your program feel comfortable using computers	Clinic staff not expected to use computers for work purposes
C12 12-step programs (AA/NA) are recommended to many of your clients	12 step programmes not accessible in most settings
C21 Behavior modification (contingency management) is used with many of your clients	Behaviour modification not implemented – no substance abuse behaviour change in PHC
C22 You have the skills needed to conduct effective individual counseling.	General staff not expected to provide individual counselling, individual counselling not widely implemented
C26 Cognitive theory (RET, RBT) guides much of your counseling.	Counselling not implemented
C31 Pharmacotherapy and related medications are important for many of your clients	Staff see not only substance abuse or mental health clients – medication will be widely used for other conditions
D11 Counselors in your program are given broad authority in treating their clients	Counselling not implemented by nursing staff
