# Supplementary material for: Adaptation of the Texas Christian University Organisational Readiness for Change Short Form (TCU-ORC-SF) for use in primary health facilities in South Africa
Source: BMJ Open. 2021 Dec 17;11(12):e047320. doi: 10.1136/bmjopen-2020-047320 (PMC8689126; doi:10.1136/bmjopen-2020-047320)
Supplement: Author's manuscript [file bmjopen-2020-047320.draft_revisions.pdf]

BMJ Open

BMJ Open is committed to open peer review. As part of this commitment we make the peer review history of every article we publish publicly available.

When an article is published we post the peer reviewers' comments and the authors' responses online. We also post the versions of the paper that were used during peer review. These are the versions that the peer review comments apply to.

The versions of the paper that follow are the versions that were submitted during the peer review process. They are not the versions of record or the final published versions. They should not be cited or distributed as the published version of this manuscript.

BMJ Open is an open access journal and the full, final, typeset and author-corrected version of record of the manuscript is available on our site with no access controls, subscription charges or pay-per-view fees (http://bmjopen.bmj.com).

If you have any questions on BMJ Open's open peer review process please email info.bmjopen@bmj.com

BMJ Open

Validation of the Texas Christian University Organisational Readiness for Change Short Form (TCU-ORC-SF) for use in primary health facilities in South Africa

Journal:	*BMJ Open*
Manuscript ID	bmjopen-2020-047320
Article Type:	Original research
Date Submitted by the Author:	01-Dec-2020

Complete List of Authors:	Brooke-Sumner, Carrie; Medical Research Council of South Africa, Alcohol Tobacco and Other Drug Research Unit;<br>Petersen-Williams, Petal; South African Medical Research Council, Alcohol, Tobacco and Other Drug Research Unit<br>Wagener, Emma; University of the Western Cape, Faculty of Community and Health Sciences<br>Sorsdahl, Katherine; University of Cape Town Faculty of Health Sciences,<br>Aarons, Gregory; University of California, San Diego, Psychiatry; University of California, San Diego<br>Myers, Bronwyn; South African Medical Research Council, Alcohol and Drug Abuse Research Unit

Keywords:	PRIMARY CARE, Organisational development < HEALTH SERVICES ADMINISTRATION & MANAGEMENT, Change management < HEALTH SERVICES ADMINISTRATION & MANAGEMENT

SCHOLARONE™
Manuscripts

BMJ

I, the Submitting Author has the right to grant and does grant on behalf of all authors of the Work (as defined in the below author licence), an exclusive licence and/or a non-exclusive licence for contributions from authors who are: i) UK Crown employees; ii) where BMJ has agreed a CC-BY licence shall apply, and/or iii) in accordance with the terms applicable for US Federal Government officers or employees acting as part of their official duties; on a worldwide, perpetual, irrevocable, royalty-free basis to BMJ Publishing Group Ltd ("BMJ") its licensees and where the relevant Journal is co-owned by BMJ to the co-owners of the Journal, to publish the Work in this journal and any other BMJ products and to exploit all rights, as set out in our licence.

The Submitting Author accepts and understands that any supply made under these terms is made by BMJ to the Submitting Author unless you are acting as an employee on behalf of your employer or a postgraduate student of an affiliated institution which is paying any applicable article publishing charge ("APC") for Open Access articles. Where the Submitting Author wishes to make the Work available on an Open Access basis (and intends to pay the relevant APC), the terms of reuse of such Open Access shall be governed by a Creative Commons licence – details of these licences and which Creative Commons licence will apply to this Work are set out in our licence referred to above.

Other than as permitted in any relevant BMJ Author's Self Archiving Policies, I confirm this Work has not been accepted for publication elsewhere, is not being considered for publication elsewhere and does not duplicate material already published. I confirm all authors consent to publication of this Work and authorise the granting of this licence.

For peer review only

Validation of the Texas Christian University Organisational Readiness for Change Short Form (TCU-ORC-SF) for use in primary health facilities in South Africa

Carrie Brooke-Sumner[1,2], Petal Petersen-Williams[1,3], Emma Wagener[4], Katherine Sorsdahl[2], Gregory A. Aarons[5,6], Bronwyn Myers[1,3]

[1] Alcohol, Tobacco and Other Drug Research Unfuit, South African Medical Research Council

[2] Alan J Flisher Centre for Public Mental Health, Department of Psychiatry and Mental Health, University of Cape Town, South Africa

[3] Division of Addiction Psychiatry, Department of Psychiatry and Mental Health, University of Cape Town, South Africa

[4] Faculty of Community and Health Sciences, University of the Western Cape, Cape Town, South Africa

[5] Department of Psychiatry, University of California, La Jolla, San Diego, CA, USA

[6] Child and Adolescent Services Research Center, San Diego, CA, USA

Corresponding Author: Carrie.Brooke-Sumner@mrc.ac.za

Abstract

Objectives: The Texas Christian University Organisational Readiness for Change Scale (TCU-ORC) assesses factors influencing adoption of evidence-based practices. It has not been validated in low- and middle-income countries (LMIC). This study assessed its psychometric properties in a South African setting with the aim of developing a shorter, pragmatic measure.

Setting: This study was conducted in 24 South African primary health care clinics in the Western Cape Province.

For peer review only - http://bmjopen.bmj.com/site/about/guidelines.xhtml

Participants: The TCU-ORC and two other measures, the Organisational Readiness to Change Assessment (ORCA) and the Checklist for Assessing Readiness for Implementation (CARI) were administered. The questionnaire was re-administered after 2 weeks to obtain data on test-retest reliability. Three hundred and ninety-five surveys were completed: 281 participants completed the first survey, and 118 re-completed the assessments.

Results: We used exploratory factor analysis (EFA) to identify latent dimensions represented in the data. Cronbach's alpha for each subscale was assessed and we examined the extent to which the subscales and total scale scores for the first and retest surveys correlated. Convergent validity was assessed by the correlation coefficient between the TCU-ORC, ORCA and CARI total scale scores. EFA resulted in a three-factor solution. The three subscales proposed are Clinic Organisational Climate (8 items), Motivational Readiness for Change (13 items), and Individual Change Efficacy (5 items) (26 items total). Cronbach's alpha for each subscale was >.80. The overall shortened scale had a test-retest correlation of r = .80, p < 0.01, acceptable convergent validity with the ORCA scale (r = .56, p < .05), moderate convergence with the CARI (r = 39, p<.05) and strong correlation with the original scale (r = 0.79, p < .05).

Conclusions: This study presents the first psychometric data on the TCU-ORC from a LMIC. The proposed shortened tool may be more feasible for use in LMICs.

Trial registration: Not applicable.

Article summary – strengths and limitations

- This study presents a pragmatic tool for assessing organisational readiness for change in primary health facilities in South Africa.

For peer review only - http://bmjopen.bmj.com/site/about/guidelines.xhtml

- Factor structure and psychometric properties including temporal stability and convergent validity are presented indicating usefulness of this tool in assessing readiness for change and enabling innovation adoption.
- The adaptation and validation of this shortened tool is a based on data collected from staff in primary health facilities in South Africa indicating its relevance for nurse-led and managed low- and middle-income primary health settings.
- Data collected reflect the sociodemographic and organisational context of health facilities in the study and contextual adaptation of the tool may be required for other settings.

Keywords

Health system resilience, organisational readiness for change, factor analysis, innovation adoption, evidence-based practice, task-sharing, primary care

Background

The treatment gap for mental disorders in South Africa, like other low- and middle-income countries (LMIC) and high-income countries (HIC), is well documented, with increasing attention being directed towards addressing this gap by providing mental health services within the context of primary care. With the reality of constrained budgets for health and human resource limitations in South Africa and globally, task-sharing of interventions to address mental disorders is gaining traction, whereby specific mental health services are delegated to non-specialist cadres with appropriate support and onward referral (1, 2). This approach is supported globally (3, 4) as a strategy for building health system resilience (5, 6) and is being investigated in a number of effectiveness and implementation studies in primary health care (PHC) settings in South Africa (2,

For peer review only - http://bmjopen.bmj.com/site/about/guidelines.xhtml

7-9). As the introduction of task-shared interventions can be complex, some interventions fail to be implemented despite a supportive policy environment (10). There may be several reasons for implementation failure in this context (11). For example, psychosocial interventions can be complex in nature (e.g. multiple sessions, psychological content); community contexts where patients live may challenge participation in sessions (e.g. poverty, violence) (12, 13); and importantly, the organisational setting of primary care facilities presents a complex environment for introducing mental health and other behavioural health innovations (e.g. heavy workloads, resource shortages, skills limitations, poor staff morale) (14-21). Innovations need to be implemented by individuals embedded in complex organisations and difficult social contexts (22-24). This may help explain why many effective interventions do not transition to sustained routine practice in primary health organisations (25, 26), and why reducing the evidence-to-practice gap remains a priority for health services research.

Organisational readiness for change (ORC) is a provides a theoretical framework for conceptualising factors supporting or hindering initial adoption of new evidence-based practices. ORC, as defined by Weiner, is a shared psychological state that relies on organisation members' motivation to change (change commitment) and belief in their own capacity to change (change efficacy) (27). Commonly identified components contributing to this shared state include organisational dynamics, climate and culture (particularly leadership from management), and individual members' characteristics and readiness to accept change, including attitudes to evidence based practices (27-31). Assessing ORC enables implementers to identify and develop approaches to reduce factors hindering innovation adoption and strengthen those factors that support adoption. In South Africa, constraints to innovation adoption include lack of resources, high workloads,

For peer review only

For peer review only - http://bmjopen.bmj.com/site/about/guidelines.xhtml

inadequate staff skills, high levels of staff turnover, and complex interpersonal and hierarchical relationships (13, 18, 32, 33). The assessment of ORC in South African settings has substantial potential to enable identification and mitigation of these constraints.

A variety of instruments for assessing ORC have been developed (34) and used in HIC. Assessment of ORC in LMICs is limited to date but may have important potential for improving implementation of quality health care. The resources, operations, and outer system and inner organizational contexts and processes are likely to differ between LMICs and HICs. This study aimed to address this research gap through contextually adapting and validating an instrument for assessing ORC in primary health settings in South Africa (35). The Texas Christian University ORC (TCU-ORC) scale (36) is among the most widely used of the ORC scales and has been tailored for use in health services research and with adaptation may be suitable for use in LMICs. Although there is good evidence for the instrument's validity in other settings (34), and it has been used in South Africa (37), little is known about the psychometric properties in LMICs and the South African context in particular. The TCU-ORC is a comprehensive measure of ORC assessing both individual psychological and organizational and health system influences on ORC. However, with 125-items, its length may make it unpractical for routine use in busy and under-resourced services within LMICs. There are continuing calls for development and testing of brief and pragmatic measures (e.g., low burden, important to stakeholders, actionable, psychometrically strong, related to theory) and such measures are more likely to be used in research and practice (38, 39).

For peer review only - http://bmjopen.bmj.com/site/about/guidelines.xhtml

This study was nested within Project MIND, a cluster randomised controlled trial evaluating two approaches to resourcing the integration of task-shared counselling for patients with depression and/or hazardous or harmful alcohol use (2). The Project MIND counselling programme comprises three-sessions of motivational interviewing and problem-solving therapy delivered by trained lay counsellors (2, 40). The integration of this service constitutes a change to the basket of health services currently available within primary care clinics, providing an opportunity for contextually validating the TCU-ORC. This study aimed to assess the psychometric properties of the TCU-ORC instrument in South African primary care clinics. Findings from this study will guide the development of a shorter version of the TCU-ORC that may be more feasible for routine use in busy primary care settings.

Methods

Methods for the Project MIND trial (2) and this psychometric validation sub-study (35) are fully described elsewhere. Briefly, the validation study involved administering a set of ORC assessments to providers within Project MINF trial sites at two time points with the purpose of establishing the internal consistency, temporal stability and construct validity of the long-form TCU-ORC questionnaire.

Participants and Procedures

Data collection took place from July to December 2018 at the 24 facilities that participated in the MIND trial. Following a workshop that introduced the study in the facilities, health service employees (managers and staff) were requested to complete a paper-based, English language self-report survey, after granting written informed consent. English is the official language of business of the South African health system.

For peer review only - http://bmjopen.bmj.com/site/about/guidelines.xhtml

Participants at workshops included facility managers, operational managers, medical officers, psychiatric nurses, general nursing staff from the chronic disease care team, and lay counsellors. We aimed to secure participation of 12 staff members from each facility (288 participants in total), however the number of participants per workshop was at the discretion of the facility manager based on staff availability. Between one and two weeks after the workshop, Project MIND fieldworkers asked a sub-sample of participants (convenience sample) from each facility to re-complete the paper-based survey comprising the ORC measures to enable assessment of test-retest reliability. These fieldworkers also approached nursing staff directly to fill in the questionnaire to gain enough responses for the first survey. In total, 395 surveys were completed: 281 participants completed the first survey and 118 participants completed both the first and test-retest survey.

Measures

The survey comprised three measures of ORC; the TCU-ORC (36), the Organisational Readiness to Change Assessment (ORCA) (41) and the Checklist for Assessing Readiness for Implementation (CARI) (42).

The four domains of the TCU-ORC (125 items total) measure Motivational Readiness for Change (Subscale A: covering staff's readiness, efficacy and support needs for implementing new practices; 33 items; e.g. clinical staff at your program needs guidance in matching client needs with services); Institutional Resources (Subscale B: adequacy of infrastructure, training and management; 31 items; e.g. much time and attention are given to staff supervision when needed);

For peer review only - http://bmjopen.bmj.com/site/about/guidelines.xhtml

Staff Attributes (Subscale C: staff skills, management planning and leadership; 31 items; e.g. learning and using new procedures are easy for you) and Organisational Climate (Subscale D: shared goals, communication structure, work environment; 30 items; e.g. the heavy staff workload reduces the effectiveness of your program). Items are scored on a five-point Likert scale ranging from strongly disagree to strongly agree. In development, these subscales showed acceptable reliability (alpha > .70 Subscale A; alpha > .60 Subscale B; alpha > .50 Subscale C; alpha > .05 Subscale D) (36).

The Organizational Readiness For Change Assessment (ORCA) (41) comprises 50 items across three scales. These assess (1) strength of evidence for the change to be introduced (9 items; e.g. the proposed change to the service delivery of the facility is supported by clinical experience with patients in other health care systems); (2) quality of the organisational context (22 items; e.g. in general in my facility, when there is agreement that change needs to happen we have the necessary support in terms of budget or financial resources) and (3) capacity for organisational facilitation of the change (19 items; e.g. communication will be maintained through regular meetings covering the intervention). Items are scored on a five-point Likert scale ranging from strongly disagree to strongly agree. There is evidence for the overall reliability and factor structure of the ORCA (alpha .74, .85 and .95 for the subscales 1, 2 and 3, respectively) (41).

The CARI checklist (42) includes 36 items that assess: (1) organisational capacity (five items; e.g. to what extent do you think technical assistance is available for the intervention being implemented); (2) organisational capacity (four items; e.g. to what extent do you think financial resources are adequate and available to introduce and sustain the intervention); (3) functional considerations (four items, e.g. to what extent do you think there is a system in place to share patient outcomes with staff and management), (4) organisational culture/climate (five items, e.g.

For peer review only - http://bmjopen.bmj.com/site/about/guidelines.xhtml

to what extent do you think there are open lines of communication in place in this facility; (5) senior leadership (five items; e.g. to what extent do you think senior leadership is convinced of the value of this intervention; (6) staff capacity (five items; e.g. to what extent do you think staff show they are ready for this type of practice change); (7) implementation plan (three items; e.g. to what extent do you think there is intent to monitor fidelity of the intervention and (8) training; (five items, e.g. to what extent do you think adequate time will be set aside for training, reflection and practice). This instrument was selected because it contains items that are broadly similar in content and wording to the TCU-ORC items although data on its psychometric properties remain to be published. Neither of the additional instruments have been validated in LMIC to our knowledge.

Twenty-three items across the four subscales of the TCU-ORC were removed prior to the assessment as they were not appropriate for the South African PHC context, (See Supplementary Table 1 for item removal based on discussion with the research group), leaving 104 items in the ORC for further assessment. Some of the removed items related to computer availability. While staff in primary care settings are meant to have access to computers and the internet, in practice this is often not the case with staff having limited access to computers where these are available. Minor editing of items was carried out to improve readability and appropriateness for clinic staff (for example ‘your program’ was edited to read ‘your facility’).

Analyses

Stata 16 statistical software was used for analysis. Cases with >50% missing data were removed (43). Normality tests were conducted for all measures using the Shapiro-Wilks test in combination with visual inspection of the normality plots. We used exploratory factor analysis, specifically Principal Axis Factoring (PAF) to identify latent dimensions represented in the variables (44, 45). The factor solution was rotated using Oblique Oblimin rotation, which is suited when factors are

For peer review only - http://bmjopen.bmj.com/site/about/guidelines.xhtml

possibly related, but measure different aspects of a construct (46). Factor extraction was determined on the basis of the theoretical structure of the TCU-ORC (36), as well as the Kaiser K0 rule for common factors, in combination with Horn's parallel analysis (47-49). Decisions around item retention were guided by item communalities and loading of items onto factors. Items with communalities of at least .70 are considered highly desirable (50). Although items with loadings greater than .40 are often retained (51), the decision was made to include items with factor loadings above .60 for parsimony due to the large number of items and to allow for a more stable factor solution.

Next, internal consistency of each subscale was assessed by calculating Cronbach's alpha of the reduced item subscales. Internal consistency is considered acceptable at values above .70 for generalisability (52). To assess temporal stability, we examined the extent to which the subscales and total scales for the first and retest surveys correlated with each other, with a correlation coefficient above .70 considered adequate. Convergent validity was assessed by calculating the correlation coefficient between the TCU-ORC, ORCA and CARI measures for the total scales (36, 41, 42).

Results

After removal of cases with less than 50% data, a total 275 participants with adequate responses who had completed the first survey and 118 participants who had completed both the first and retest survey were included in the analyses. Participants' sociodemographic characteristics are summarized in Table 1. The sample comprised predominantly female nursing staff, reflecting the staffing profile of primary clinics in South Africa. A large proportion had worked at their current

For peer review only - http://bmjopen.bmj.com/site/about/guidelines.xhtml

facility for more than five years and the majority had been in their profession for more than five years (Table 1).

Exploratory factor analysis

Assumption testing for factor analysis

Normality of the TCU-ORC was established by a non-significant Shapiro-Wilk result (p = .11) and visual inspection of the Histogram and the Q-Q plot. Bartlett's test of sphericity indicated that correlations between items were sufficiently large for factor analysis ($\chi^2(5356) = 12099.13$, $p < .01$). The Kaiser-Meyer-Olkin (KMO) measure of sampling adequacy was found to be acceptable with a value of .70 (53).

Factor extraction. The initial extraction was run with the Kaiser rule specified, retaining factors with eigenvalues greater than 1. This identified 74 factors. A parallel analysis (PA) was run in Stata, using Glorefeld's (1995) extension and using the 99th percentile to compare eigenvalues in order to increase sensitivity (49). This revealed 6 factors that had adjusted eigenvalues greater than those randomly generated at a 10% margin (54).

When conducting a PA, factors with a large number of variables are prone to inaccuracies due to poorly defined factors (54). As this measure contains a large number of variables, it is likely that the results of the PA over extracted factors. The decision was made to specify four factors, according to these results and the theoretical basis of the original TCU-ORC (36). After the initial extraction of four factors, we observed that multiple items from two subscales loaded onto one factor. Visual inspection of the scree plot confirmed three distinct points above the point of inflection (55), suggesting a three-factor solution. Subsequent analyses were based on the specification of a three-factor solution.

For peer review only - http://bmjopen.bmj.com/site/about/guidelines.xhtml

Factor loadings of the final factor structure are presented in Table 2. Eleven extractions were performed overall to reach the final three factor solution. This explained 72.4% of shared variance. We removed items with cross-loadings of .3 or above on any item and items were removed one at a time in sequential factor analysis runs (51). Sampling adequacy using the KMO Measure was assessed for each item as a post-estimation after each extraction. In each extraction, items with loadings less than .60 were removed after assessing how removing items affected the Cronbach's alpha for each subscale and whether internal consistency improved. The final three factor solution consists of 26 items, having removed 99 items and therefore providing a simplified, shortened version of the scale (Table 3).

Factor 1 'Clinic Organisational Climate' consists of 13 items, with loadings between .61 and .72. This factor is made up of items from the original Institutional Resources, Staff Attributes and Organizational Climate subscales. The revised subscale had a Cronbach's alpha of .90 and correlated with the original 'Organizational Climate' subscale ($r = .82$, $p < .05$).

Factor 2 'Motivational Readiness for Change' consists of eight items, with loadings between .64 and .78. The items in this subscale were all originally in the TCU-ORC's Motivational Readiness for Change subscale. This subscale had good reliability with a Cronbach's alpha of .93 and correlated with the original 'Motivational Readiness for Change' subscale ($\alpha = .79$).

Factor 3 'Individual Change Efficacy' consists of five items, with factor loadings between .62 and .76. The items in this subscale all originated from the 'Staff Attributes' subscale. This subscale had a Cronbach's alpha of .83 and correlated with the original 'Individual Change Efficacy' subscale ($\alpha =.57$).

For peer review only - http://bmjopen.bmj.com/site/about/guidelines.xhtml

Overall, the 26-item version of the TCU-ORC was strongly correlated with the original scale (r = .79, $p < .05$).

Temporal stability

A test-retest correlation coefficient between the initial and follow-up versions of the TCU-ORC was conducted, using only those items retained after PAF for both administration versions. Temporal stability was assessed for the overall scale as well as for each sub-scale. The correlation results are presented in Table 4. Subscales show adequate temporal stability for Clinic Organisational Climate (r = .87, $p < .01$), Motivational Readiness for Change (r = .60, $p < .01$), with exception of the Individual Change Efficacy subscale (r = .53, $p < .01$), Overall, the shortened scale was found to have good consistency across time with a test-retest correlation of r = .80, $p < .01$.

Convergent validity

The reliability coefficients of criterion measures were good for the ORCA (α = .96) and CARI (α = .96), supporting the interpretation of convergent correlations. The shortened version of the TCU-ORC was found to have acceptable convergent validity with the ORCA scale (r = .56, $p < .05$), with a moderate correlation with the CARI (r = .39, $p < .05$). Convergent validities of r = .70 are considered ideal, with validities less than r = .50 considered insufficient in organisational research (56) (54).

Discussion

For peer review only - http://bmjopen.bmj.com/site/about/guidelines.xhtml

Validated measures for assessing organizational functioning and dynamics in health organisations in LMICs are currently lacking (57). These measures are needed to assist health systems as they grapple with the need to be resilient and responsive to the changing health needs of the population (6, 32). To be applicable and useful, measures must be pragmatic, and useful to the service providers involved in delivering and managing systems and facilities (38, 39). As a component of health systems strengthening, it is critical to assess and understand organizational readiness for change with the view to implementing evidence-based practices. This study presents findings on the psychometric properties of the TCU-ORC amongst a sample of health workers from primary care settings in the Western Cape Province of South Africa. While the usefulness of this measure has been investigated by studies from a number of HIC (e.g. (58, 59)), this is the first investigation of its kind in primary care in South Africa and to the best of our knowledge, in other LMICs.

The overall goal of this work was to develop a contextually relevant and validated measure, feasible for use in primary care within South Africa (35). While the original measure has been widely used in better resourced settings, 125 items is not a feasible for use as a routine assessment for ORC within the busy and under-resourced South African health system or health settings in other LMICs. A shorter, pragmatic instrument capable of collecting essential information to inform implementation is required for completion by primary care staff with high workloads and multiple competing priorities (13) who report lacking time to complete questionnaires. The need for reducing response burden and respondent fatigue, is aligned with current thinking among global mental health researchers who have noted response burden as a threat to the quality of data collected by lengthy measures (60). The current study resulted in the reduction of the TCU-ORC to 26 items. While this is a considerable reduction, further shortening may be needed to produce a scale truly feasible for routine use in primary care within LMICs. Experience from HIC settings

For peer review only

For peer review only - http://bmjopen.bmj.com/site/about/guidelines.xhtml

underscores the importance of ensuring implementation support measures are responsive to stakeholders' needs, present a low burden and have wide application across a multitude of settings (38, 39, 61). Scales with large number of items have been shown to be feasibly shortened to as low as 3 items per subscale retaining high degree of validity and reliability (alpha > .9) e.g. (62). With this in mind, we plan to conduct a Delphi study with stakeholders and experts from the South African primary care arena to further shorten the tool and enable integration of stakeholder perspectives.

Factor analysis showed overlap between three of the original domains, suggesting a three-factor structure for the South African data, in contrast to the original four domains (Motivational Readiness for Change (A), Institutional Resources (B) Staff Attributes (C) and Organisational Climate (D)). The proposed domains for the South African data, as shown in Table 2 are (1) Clinic organisational climate, (13 items) (2) Pressures for change (8 items) and (3) Individual change efficacy (5 items). Each domain and the overall scale showed strong internal consistency, adequate temporal stability, and moderate convergent validity with the ORCA and CARI measures. The temporal stability for the Individual Change Efficacy scale may reflect the unstable and uncertain climate within which health providers often operate. At the time of this study, many of the clinics were affected by community violence and service delivery protests that sometimes led to clinic closures or restricted service delivery. It is plausible that these contextual factors may have impacted on individual providers perceptions of change efficacy, especially as efficacy for change is often context dependent. However, more research is needed to identify and address such "bridging factors" that demonstrate bi-directional influences of outer system and inner organizational contexts (63).

For peer review only - http://bmjopen.bmj.com/site/about/guidelines.xhtml

For peer review only

Three items with cross loadings >.3 (.3 to .5) were removed suggesting retained items were representing relatively unique constructs that were understood by respondents (57). The domain 'Clinic Organisational Climate' incorporates items from the Institutional Resources, Staff Attributes and Organisational Climates domains of the original TCU-ORC. This may be because institutional resources, staff attributes, and trusting supervisory relationships become essential prerequisites for staff's functioning within constrained environments. These are perceived as key aspects of the organizational climate and have been previously shown to group as elements of organisational functioning (21). Thirteen items were retained in this domain suggesting these are key to assessing organizational climate in South African PHC settings. Items included those relating to management, supervision, teamwork, leadership and trusting relationships, which aligns with other work investigating primary care functioning in South Africa (18, 32, 64, 65). Findings from qualitative work assessing primary care facilities' preparedness for mental health counselling implementation also suggest intersecting relationships between resource availability, management style and facility environment (20). The Pressures for Change domain remained largely consistent with the corresponding domain of the original TCU-ORC. Only a small number of items were retained from the C domain on Staff Attributes - these related to individual agency for change. Organisational theory outlines how individuals within an organisation can be resistant to change, particularly in the context of heavy workloads (27, 66) known to be present in South African facilities. South African facility managers are often clinicians, without management training, who work in high workload environments and indicate that workload constrains innovation adoption (67). The South African health system has a strong hierarchy of downward supervision and management, compliance and targets and this may hinder frontline managers and staff from being responsive to patient needs and public health burden (68). At the same time, nurses are the majority

For peer review only - http://bmjopen.bmj.com/site/about/guidelines.xhtml

of clinic staff and are key to organisational climate and change. The items retained in this scale may therefore be most relevant for assessing individual perceptions amongst nursing staff on their agency and willingness to change.

In relation to convergent validity, correlation of TCU-ORC-SF with the ORCA showed large effect, but with the CARI correlation showed medium effect. These two additional instruments were also designed to assess organisational readiness for change in HICs, and although some aspects of organisational culture, dynamics and functioning are translatable to other settings, others may not be robustly relevant in the South African PHC context. With regards to correlation of the shortened tool with the CARI, it is likely that the restricted range of responses in the CARI resulted in a lower correlation (69). The CARI items are measured on a 4-point scale, while the TCU-ORC items are measured on a 5-point scale. Nonetheless, it is the opinion of the researcher/s that these validity correlations between the ORCA, CARI and TCU-ORC scales are acceptably large to assume convergence. We propose that the revised domains proposed here more closely capture the essentials of organisational readiness for change in South African PHC settings than the original measures.

Limitations

Data for this study were collected from primary care facilities from the Western Cape province. This province is more well-resourced than other provinces. Data from the study showed a sociodemographic profile of facility staff that reflects the qualifications, professions and work experience common across primary care settings in the country. This suggests appropriateness for the broader South African context, although there are important disparities in human and other resources and variations in burden of disease and contexts across provinces (5, 70). The survey was in English which is not the first language of health providers in some settings, however English

For peer review only - http://bmjopen.bmj.com/site/about/guidelines.xhtml

is the official business language of government agencies and health providers would be expected to be proficient. The ORCA and CARI measures have also not been adapted for use in LMIC use so there may be limitations in their validity for this context which may have influenced convergent validity findings.

Conclusion

This study has presented validity data and contextual adaptation of the TCU-ORC in a LMIC setting. Based on the findings from this study, we propose a short form of the TCU-ORC, comprising 26 of 125 items. The three subscales proposed: Clinic Organisational Climate, Motivational Readiness for Change, and Individual Change Efficacy contain key items for assessing ORC in this context. This scale may be useful as a tool for health service managers to highlight potential roadblocks to the introduction of innovations that may need to be addressed when developing an implementation strategy. This shortened tool however may still be too lengthy for widespread practical use in primary care services and further shortening involving stakeholders who will use the tool in practice is required.

Declarations

Ethics approval and consent to participate

Ethical approval was granted for this study by the Human Research Ethics Committee of the South African Medical Research Council (EC004-2-2015) and approval for working in facilities was also granted by the Western Cape Provincial Department of Health.

Consent for publication

For peer review only - http://bmjopen.bmj.com/site/about/guidelines.xhtml

Not applicable.

Patient and public involvement

It was not appropriate to involve patients or the public in this study since it focused on primary health staff as employees of the health system. We will present the shortened tool to staff and managers of the study facilities as part of the post-trial engagement meetings for Project MIND.

Availability of data and materials

Data from the study may be made available upon reasonable request.

Competing interests

The authors declare that they have no competing interests.

Funding statement

This study is funded jointly by the British Medical Research Council, Wellcome Trust, Department for International Development, the Economic and Social Research Council, the Global Challenges Research Fund (MR/M014290/1) as well as funding from the South African Medical Research Council (SAMRC, contract). CBS was supported as a postdoctoral fellow by funding from the SAMRC through its Division of Research Capacity Development under the Intra-Mural Postdoctoral Fellowship Programme from funding received from the South African National Treasury. The content hereof is the sole responsibility of the authors and does not necessarily represent the official views of the SAMRC or the funders.

Author contributions

CBS and BM conceptualised the study. CBS and PPW managed data collection. BM and KS are Principal Investigators on the Project MIND trial in which the study is nested. CBS and EW

For peer review only

For peer review only - http://bmjopen.bmj.com/site/about/guidelines.xhtml

conducted factor analysis. EW provided statistical technical input and drafted some sections of the results. GA provided technical guidance on methods and analysis. All authors reviewed and contributed to the final version of the manuscript.

Acknowledgements

The authors thank Lesley-Anne Erasmus-Claasen, Yuche Jacobs and Nombuso Moshiga for their fieldwork on the study as well as all participating facilities, managers and staff.

For peer review only

For peer review only - http://bmjopen.bmj.com/site/about/guidelines.xhtml

429

430 **Table 1 Sociodemographic data from PHC facility-based staff**

Item	Category	Frequency	Percentage
Gender	Female	214	88.4%
	Male	28	11.6%
Age	20-30	14	5.3%
	31-40	72	27.2%
	41-50	73	27.5%
	51-60	84	31.7%
	61-65	22	8.3%
Ethnicity	Black African	136	50.2%
	Coloured	117	43.2%
	White	13	4.8%
	Indian/Asian	3	1.1%
	Other	2	0.7%
Time at facility	0-6 months	22	8.1%
	6-11 months	20	7.4%
	1-3 years	61	22.5%
	3-5 years	48	17.7%
	More than 5 years	120	44.3%
Education level	Completed high school	62	23.0%
	Some tertiary education	65	24.1%
	Bachelor's degree	51	18.9%
	Master's degree	7	2.6%
	Doctoral degree	3	1.1%
	Diploma in healthcare	74	27.3%
	Other	8	3.0%
Profession*	Medicine	25	9.1%
	Social work	6	2.1%
	Counselling	62	22.7%
	Psychology	2	0.7%
	Nursing	149	54.8%
	Administration	30	11.0%
	Other	18	6.6%
Time in profession	0-6 months	7	2.6%
	6-11 months	7	2.6%
	1-3 years	36	13.4%
	3-5 years	25	9.4%
	More than 5 years	192	72.0%

431 *Some participants selected working in more than one profession

For peer review only - http://bmjopen.bmj.com/site/about/guidelines.xhtml

For peer review only

432 **Table 2 Factor analysis by domains of the original TCU-ORC**

For peer review only

For peer review only - http://bmjopen.bmj.com/site/about/guidelines.xhtml

Subscale A: Motivational Readiness for Change (29 items included in factor analysis)

Subscale B: Institutional Resources (22 items included in factor analysis)

Subscale C: Staff Attributes (24 items included in factor analysis)

Subscale D: Organisational Climate (29 items included in factor analysis)

Initial Factor 1		Final factor 1<br>Clinic Organisational Climate		Initial Factor 2		Final factor 2<br>Motivational Readiness for Change		Initial Factor 3		Final Factor 3<br>Individual Change Efficacy		Initial Factor 4	
Items	Loadings	Items	Loadings	Items	Loadings	Items	Loadings	Items	Loadings	Items	Loadings	Items	Loadings
A26	.32	B1	.66	A1	.56	A1	.68	B20	.35	C8	.68	B2	.38
B1	.64	B3	.61	A2	.51	A3	.77	C1	.44	C9	.71	C24	.41
B3	.53	B10	.68	A3	.55	A4	.78	C2	.30	C10	.76	D5	.36
B4	.39	B16	.71	A4	.59	A5	.77	C4	.48	C11	.62	D12	.35
B5	.56	B19	.65	A5	.63	A6	.73	C8	.50	C18	.62	D15	.34
B6	.51	C7	.66	A6	.59	A7	.70	C9	.61			D16	.34
B7	.60	D7	.62	A7	.57	A8	.70	C10	.52			D19	.44
B8	.59	D11	.65	A8	.58	A10	.64	C11	.47			D22	.54
B10	.69	D14	.66	A9	.61			C12	.34			D27	.33
B11	.64	D21	.70	A10	.69			C13	.31				
B12	.39	D25	.72	A11	.63			C14	.35				
B13	.44	D26	.66	A12	.64			C17	.44				
B14	.48	D28	.64	A13	.56			C18	.53				
B15	.39	D29	.61	A14	.55			C23	.45				
B16	.70			A15	.60			D6	.33				

For peer review only - http://bmjopen.bmj.com/site/about/guidelines.xhtml

B17	.58	A16	.59
B18	.52	A18	.30
B19	.62	A19	.50
B21	.45	A20	.46
C3	.44	A21	.49
C5	.34	A22	.37
C6	.53	A23	.51
C7	.65	A24	.34
C10	.33	A28	.38
C13	.32	A29	.30
C14	.49	C2	.31
C15	.35	C8	.36
C20	.38	C10	.34
C21	.41	C16	.40
C23	.39	C17	.32
D3	.42	C18	.43
D4	.53	D6	.45
D5	.62	D15	.32
D7	.61	D24	.36
D8	.67		
D9	.64		
D11	.60		
D13	.56		
D14	.67		

For peer review only

D16	.40
D17	.50
D18	.55
D20	.45
D21	.67
D23	.45
D25	.70
D26	.63
D28	.61
D29	.61

Extraction Method: Factor Analysis.
Rotation Method: Oblique Oblimin

For peer review only

Table 3 Excluded items and proposed revised domains*

Original TCU ORC domain	Number of items in initial factor analysis<br><br>(104 total)	Excluded items<br><br>(loadings < .6 through 11 extractions)	Proposed revised domains	Number of items in proposed scale
A Motivational Readiness for Change	29	Your facility needs guidance in<br><br>A2 using patient assessments to guide<br><br>clinical care<br><br>A9 defining its mission<br><br>A11 assigning or clarifying staff roles<br><br>A12 setting out accurate job descriptions for staff<br><br>A13 evaluating staff performance<br><br>A14 improving relations among staff<br><br>A15 improving communications among staff<br><br>A16 improving record keeping and information systems<br><br>A17 basic computer skills/programs<br><br>A18 specialized computer applications<br><br>A19 new developments and practices in your area of responsibility<br><br>A20 new procedures being used or planned<br><br>A21 continuous professional development<br><br>A22 new laws or regulations you need to know about<br><br>A23 management or supervisory responsibilities<br><br>Current pressures to make facility changes come from<br><br>A24 community members (patients)<br><br>A25 other staff members<br><br>A26 facility supervisors or managers<br><br>A27 district level managers<br><br>A28 community groups<br><br>A29 national and provincial DOH structures	**Motivational Readiness for Change**<br><br>Clinical staff at your facility needs guidance in:<br><br>A1 assessing patient needs<br><br>A3 using patient assessments to document patient improvements<br><br>A4 matching patient needs with services<br><br>A5 improving relationship with patients<br><br>A6 improving patient thinking and problem-solving skills to manage chronic disease<br><br>A7 improving behavioural management of patients<br><br>A8 identifying and using evidence-based practices<br><br>Your facility needs guidance in:<br><br>A10 setting specific goals for improving services	8

For peer review only - http://bmjopen.bmj.com/site/about/guidelines.xhtml

B Institutional Resources	22	B2 Frequent staff turnover is a problem for your facility<br>B4 The space in your facilities are adequate for conducting group or individual counselling<br>B5 You have clinical supervisors who are capable and qualified<br>B6 You learned new skills or techniques at a professional training in the past year<br>B7 Much time and attention are given to staff supervision when needed<br>B8 Staff in your facility are able to spend the time needed with patients<br>B9 Equipment at your facility is mostly old and outdated<br>B11 Support staff in your facility have the skills they need to do their jobs<br>B12 Offices in your facility allow the privacy needed for individual counselling<br>B13 Your facility holds regular in-service training<br>B14 Your facility has enough staff to meet current patient needs<br>B15 Clinical staff in your program are well-trained<br>B17 Offices and equipment in your facility are adequate<br>B18 Your facility provides a comfortable reception/waiting area for patients<br>B20 A larger support staff is needed to help meet needs at your program.<br>B21 Staff in your facility are able to attend professional training<br>B22 Staff concerns are ignored in most decisions made in your facility<br>D29 You feel encouraged to try new and different techniques	**Clinic organisational climate**<br>B1 You have good facility management at your facility<br>B3 Staff training and continuing education are priorities in your facility<br>B10 Clinical and management decisions for your facility are well planned<br>B16 You have confidence in how decisions at your facility are made<br>B19 Staff meet frequently with clinical supervisors about patient needs and progress<br>C7 Your facility encourages and supports professional growth<br>D7 Ideas and suggestions in your facility get fair consideration by management<br>D11 Your facility staff is always kept well informed<br>D14 Your facility operates with clear goals and objectives<br>D21 The formal and informal communication channels in your facility work very well<br>D25 Management fully trusts professional judgments of staff in your facility<br>D26 Staff members always feel free to ask questions and express concerns in your facility<br>D28 Management for your facility has a clear plan for its future	13
C Staff Attributes	24	C1 You have the skills needed to effectively perform your role<br>C2 Other staff often ask your advice<br>C3 You are satisfied with your present job	**Individual change efficacy**<br>C8 You are effective and confident in doing your job<br>C9 You are able to adapt quickly when you have to make changes	5

For peer review only - http://bmjopen.bmj.com/site/about/guidelines.xhtml

		C4 Learning and using new procedures are easy for you<br><br>C5 You are considered an experienced source of advice about services and/or facility management<br><br>C6 You feel appreciated for the job you do at work<br>C12 You regularly influence the decisions<br><br>of other staff you work with<br><br>C13 You usually accomplish whatever you set your mind on<br><br>C14 You do a good job of regularly updating<br><br>and improving your skills<br><br>C15 You regularly read professional articles<br><br>or books related to your work<br><br>C16 Other staff often ask for your opinions<br><br>about issues in the facility<br><br>C17 You are willing to try new ideas even if some staff members are reluctant<br><br>C19 You are sometimes too cautious or slow to make changes<br><br>C20 You are proud to tell others where you work<br><br>C21 You like the people you work with<br><br>C22 You are viewed as a leader by the staff you work with<br><br>C23 You consistently plan ahead and carry out your plans<br><br>C24 You would like to find a job somewhere else	C10 Keeping your professional skills up-to-date is a priority for you<br><br>C11 You give high value to the work you do<br><br>C18 You frequently share your knowledge of new ideas with others	
D Organisational Climate	29	D1 Some staff members seem confused about the main goals for your facility<br><br>D2 The heavy staff workload reduces the effectiveness of your facility<br><br>D3 You frequently hear good ideas from other staff for improving services<br><br>D4 Care plans and decisions for patients in your facility often get revised by a clinical supervisor<br><br>D5 The general attitude in your facility is to accept new and changing technology or procedures<br><br>D6 More open discussions about facility issues are needed where you work		

For peer review only

For peer review only - http://bmjopen.bmj.com/site/about/guidelines.xhtml

		D8 Staff members at your facility work together as a team<br><br>D9 Your duties are clearly related to the goals for your facility<br><br>D10 You are under too many pressures to do your job effectively<br><br>D12 New ways of working from staff are discouraged where you work<br><br>D13 Mutual trust and cooperation among staff in your program are strong<br><br>D15 Staff members at your facility often show signs of high stress and strain<br><br>D16 It is easy to change procedures at your facility to meet new conditions<br><br>D17 Staff in your facility can try out<br><br>different techniques to improve their effectiveness<br><br>D18 Staff members at your facility get along very well<br><br>D19 Staff members are given too many rules in your facility<br><br>D20 Staff members at your program are quick<br><br>to help one another when needed<br><br>D22 There is too much friction among staff members you work with<br><br>D23 Staff members at your facility understand the work of the facility fits as part of the health improvement in your community<br><br>D24 Some staff in your facility do not do their fair share of work<br><br>D27 Staff frustration is common where you work		

* 'A' items refer to the Motivational Readiness for Change scale, 'B' items refer to the Institutional Resources scale, 'C' items refer to the Staff Attributes scale, 'D' items refer to the Organisational Climate scale.

For peer review only - http://bmjopen.bmj.com/site/about/guidelines.xhtml

Table 4 Internal consistency and retest reliability of the new subscales

Subscale/ Factor	Description	Cronbach's Alpha	Temporal stability (*r*)
Clinic Organizational Climate	Management practices, trust, leadership, communication, appreciation of staff, facility resources, attitudes to change	.90	.87**
Motivational Readiness for Change	Areas of need for facility improvement, and staff development	.91	.60**
Individual Change Efficacy	Individual self-efficacy, adaptability, knowledge and skills	.83	.53**
Total revised scale		.86	.80**

** Significant at the .01 level

References

1. Department of Health RoSA. National Mental Health Policy Framework and Strategic Plan 2013-2020. Pretoria: Department of Health South Africa, 2013.
2. Myers B, Lund C, Lombard C, Joska J, Levitt N, Butler C, et al. Comparing dedicated and designated models of integrating mental health into chronic disease care: study protocol for a cluster randomized controlled trial. Trials. 2018;19(1):185.
3. De Silva MJ, Ryan G. Global mental health in 2015: 95% implementation. The lancet Psychiatry. 2016;3(1):15-7.
4. Ciapponi A, Lewin S, Herrera CA, Opiyo N, Pantoja T, Paulsen E, et al. Delivery arrangements for health systems in low-income countries: an overview of systematic reviews. Cochrane Database Syst Rev. 2017;9:Cd011083.
5. Fusheini A, Eyles J. Achieving universal health coverage in South Africa through a district health system approach: conflicting ideologies of health care provision. BMC health services research. 2016;16(1):558-.
6. Barasa EW, Cloete K, Gilson L. From bouncing back, to nurturing emergence: reframing the concept of resilience in health systems strengthening. Health policy and planning. 2017;32(suppl_3):iii91-iii4.
7. Lund C, Alem A, Schneider M, Hanlon C, Ahrens J, Bandawe C, et al. Generating evidence to narrow the treatment gap for mental disorders in sub-Saharan Africa: rationale, overview and methods of AFFIRM. Epidemiol Psychiatr Sci. 2015;24(3):233-40.

For peer review only - http://bmjopen.bmj.com/site/about/guidelines.xhtml

8. Fairall L, Petersen I, Zani B, Folb N, Georgeu-Pepper D, Selohilwe O, et al. Collaborative care for the detection and management of depression among adults receiving antiretroviral therapy in South Africa: study protocol for the CobALT randomised controlled trial. Trials. 2018;19(1):193.
9. Petersen I, Bhana A, Fairall LR, Selohilwe O, Kathree T, Baron EC, et al. Evaluation of a collaborative care model for integrated primary care of common mental disorders comorbid with chronic conditions in South Africa. BMC psychiatry. 2019;19(1):107.
10. Hanson K, Cleary S, Schneider H, Tantivess S, Gilson L. Scaling up health policies and services in low- and middle-income settings. BMC health services research. 2010;10 Suppl 1:I1.
11. Jacobs SR, Weiner BJ, Reeve BB, Hofmann DA, Christian M, Weinberger M. Determining the predictors of innovation implementation in healthcare: a quantitative analysis of implementation effectiveness. BMC health services research. 2015;15:6.
12. Eyles J, Harris B, Fried J, Govender V, Munyewende P. Endurance, resistance and resilience in the South African health care system: case studies to demonstrate mechanisms of coping within a constrained system. BMC health services research. 2015;15(1):432.
13. Brooke-Sumner C, Petersen-Williams P, Kruger J, Mahomed H, Myers B. 'Doing more with less': a qualitative investigation of perceptions of South African health service managers on implementation of health innovations. Health policy and planning. 2019;34(2):132-40.
14. Lehmann U, Gilson L. Actor interfaces and practices of power in a community health worker programme: a South African study of unintended policy outcomes. Health policy and planning. 2013;28(4):358-66.
15. Gopinathan U, Lewin S, Glenton C. Implementing large-scale programmes to optimise the health workforce in low- and middle-income settings: a multicountry case study synthesis. Tropical medicine & international health : TM & IH. 2014;19(12):1437-56.
16. Innis J, Dryden-Palmer K, Perreira T, Berta W. How do health care organizations take on best practices? A scoping literature review. Int J Evid Based Healthc. 2015;13(4):254-72.
17. Freeman M. Global mental health in low and middle income, especially African countries. Epidemiol Psychiatr Sci. 2016;25(6):503-5.
18. Gilson L, Barasa E, Nxumalo N, Cleary S, Goudge J, Molyneux S, et al. Everyday resilience in district health systems: emerging insights from the front lines in Kenya and South Africa. BMJ global health. 2017;2(2):e000224.
19. Mbau R, Gilson L. Influence of organisational culture on the implementation of health sector reforms in low- and middle-income countries: a qualitative interpretive review. Global health action. 2018;11(1):1462579.
20. Oboirien K, Harris B, Goudge J, Eyles J. Implementation of district-based clinical specialist teams in South Africa: Analysing a new role in a transforming system. BMC health services research. 2018;18(1):600.
21. Myers B, Breuer E, Lund C, Petersen Williams P, van der Westhuizen C, Brooke-Sumner C, et al. Assessing capability for implementing mental health counselling within primary care facilities in a middle-income country: A feasibility study. Journal of psychiatric and mental health nursing. 2019;26(5-6):163-74.
22. Lund C, Brooke-Sumner C, Baingana F, Baron EC, Breuer E, Chandra P, et al. Social determinants of mental disorders and the Sustainable Development Goals: a systematic review of reviews. The Lancet Psychiatry.5(4):357-69.
23. Aarons GA, Hurlburt M, Horwitz SM. Advancing a conceptual model of evidence-based practice implementation in public service sectors. Adm Policy Ment Hlth. 2011;38.
24. Cresswell K, Sheikh A. Organizational issues in the implementation and adoption of health information technology innovations: an interpretative review. Int J Med Inform. 2013;82(5):e73-86.

For peer review only - http://bmjopen.bmj.com/site/about/guidelines.xhtml

25. Lau R, Stevenson F, Ong BN, Dziedzic K, Treweek S, Eldridge S, et al. Achieving change in primary care--effectiveness of strategies for improving implementation of complex interventions: systematic review of reviews. BMJ open. 2015;5(12):e009993.
26. Lau R, Stevenson F, Ong BN, Dziedzic K, Treweek S, Eldridge S, et al. Achieving change in primary care--causes of the evidence to practice gap: systematic reviews of reviews. Implementation Science. 2016;11:1-39.
27. Weiner BJ. A theory of organizational readiness for change. Implementation Science: IS. 2009;4:67-.
28. Aarons GA. Measuring provider attitudes toward evidence-based practice: consideration of organizational context and individual differences. Child and adolescent psychiatric clinics of North America. 2005;14(2):255-71, viii.
29. Aarons GA, Cafri G, Lugo L, Sawitzky A. Expanding the domains of attitudes towards evidence-based practice: the evidence based practice attitude scale-50. Adm Policy Ment Health. 2012;39(5):331-40.
30. Attieh R, Gagnon MP, Estabrooks CA, Legare F, Ouimet M, Roch G, et al. Organizational readiness for knowledge translation in chronic care: a review of theoretical components. Implementation science : IS. 2013;8:138.
31. Williams B, Perillo S, Brown T. What are the factors of organisational culture in health care settings that act as barriers to the implementation of evidence-based practice? A scoping review. Nurse education today. 2015;35(2):e34-41.
32. Gilson L. Trust and the development of health care as a social institution. Soc Sci Med. 2003;56(7):1453-68.
33. Gilson L, Palmer N, Schneider H. Trust and health worker performance: exploring a conceptual framework using South African evidence. Social Science & Medicine (1982). 2005;61(7):1418-29.
34. Gagnon M-P, Attieh R, Ghandour EK, Légaré F, Ouimet M, Estabrooks CA, et al. A Systematic Review of Instruments to Assess Organizational Readiness for Knowledge Translation in Health Care. PloS one. 2014;9(12):e114338.
35. Brooke-Sumner C, Sorsdahl K, Lombard C, Petersen-Williams P, Myers B. Protocol for development and validation of a context-appropriate tool for assessing organisational readiness for change in primary health clinics in South Africa. BMJ open. 2018;8(4).
36. Lehman WE, Greener JM, Simpson DD. Assessing organizational readiness for change. Journal of substance abuse treatment. 2002;22(4):197-209.
37. Bowles S, Louw J, Myers B. Perceptions of organizational functioning in substance abuse treatment facilities in South Africa. International journal of mental health and addiction. 2011;9(3):308-19.
38. Glasgow RE, Riley WT. Pragmatic measures: what they are and why we need them. Am J Prev Med. 2013;45(2):237-43.
39. Glasgow RE. What does it mean to be pragmatic? Pragmatic methods, measures, and models to facilitate research translation. Health Educ Behav. 2013;40(3):257-65.
40. Sorsdahl K, Naledi T, Lund C, Levitt NS, Joska JA, Stein DJ, et al. Integration of mental health counselling into chronic disease services at the primary health care level: Formative research on dedicated versus designated strategies in the Western Cape, South Africa. 2020:1355819620954232.
41. Helfrich CD, Li YF, Sharp ND, Sales AE. Organizational readiness to change assessment (ORCA): development of an instrument based on the Promoting Action on Research in Health Services (PARIHS) framework. Implementation science : IS. 2009;4:38.
42. M. B. Checklist to assess organizational readines s (CARI) for EIP Implementation.

. 2011.

For peer review only - http://bmjopen.bmj.com/site/about/guidelines.xhtml

43. Van den Broeck J, Argeseanu Cunningham S, Eeckels R, Herbst K. Data Cleaning: Detecting, Diagnosing, and Editing Data Abnormalities. PLOS Medicine. 2005;2(10):e267.
44. Henson R, Roberts J. Use of Exploratory Factor Analysis in Published ResearchCommon Errors and Some Comment on Improved Practice. Educational and Psychological Measurement - EDUC PSYCHOL MEAS. 2006;66:393-416.
45. Mooi E, Sarstedt, M., & Mooi-Reci, I. . Principal component and factor analysis. Market Research. Singapore: Springer; 2018. p. pp 265-311.
46. Fabrigar LR, Wegener DT, MacCallum RC, Strahan EJ. Evaluating the use of exploratory factor analysis in psychological research. Psychological Methods. 1999;4(3):272-99.
47. Ledesma RD, Valero-Mora, P. Determining the number of factors to retain in EFA: An easy-to-use computer program for carrying out parallel analysis. Practical assessment, research, and evaluation. 2007;12(1):2.
48. Hayton JC, Allen DG, Scarpello V. Factor Retention Decisions in Exploratory Factor Analysis: A Tutorial on Parallel Analysis. Organizational Research Methods. 2004;7(2):191-205.
49. Dinno A. Implementing Horn's Parallel Analysis for Principal Component Analysis and Factor Analysis. Stata Journal. 2009;9:291-8.
50. Field A. Disscovering statistics using IMB SPSS statistics: Thousand Oaks: SAGE Publications Inc; 2013.
51. Costello AB, Osborne J. Best Practices in Exploratory Factor Analysis: Four Recommendations for Getting the Most From Your Analysis. Practical Assessment, Research & Evaluation. 2005;10:1-9.
52. Bartholomew DJ, Knott, M., & Moustaki, I. Latent variable models and factor analysis: A unified approach: John Wiley & Sons.; 2011.
53. Pett MA, Lackey, N. R., & Sullivan, J. J. Making sense of factor analysis. Thousand Oaks: Sage Publications; 2003.
54. Jones JM. The Influence of a Proposed Margin Criterion on the Accuracy of Parallel Analysis in Conditions Engendering Underextraction

Masters Theses & Specialist Projects: https://digitalcommons.wku.edu/theses/2446; 2018.
55. Cattell RB. The Scree Test For The Number Of Factors. Multivariate Behavioral Research. 1966;1(2):245-76.
56. Carlson KD, Herdman, A. O. Understanding the impact of convergent validity on research results. Organizational Research Methods. 2012;15(1):17-32.
57. Uy J, Macarayan EK, Ratcliffe HL, Miller K, Otupiri E, Awoonor-Williams JK, et al. Preliminary validation of the PRImary care facility Management Evaluation tool (PRIME-Tool), a national facility management survey implemented in Ghana. BMC health services research. 2019;19(1):937.
58. Rampazzo L, De Angeli M, Serpelloni G, Simpson DD, Flynn PM. Italian Survey of Organizational Functioning and Readiness for Change: A Cross-Cultural Transfer of Treatment Assessment Strategies. 12: Karger AG. p. 176-81.
59. Rowan-Szal GA, Greener JM, Joe GW, Simpson DD. Assessing program needs and planning change. Journal of substance abuse treatment. 2007;33(2):121-9.
60. Haroz EE, Kane JC, Nguyen AJ, Bass JK, Murray LK, Bolton P. When less is more: reducing redundancy in mental health and psychosocial instruments using Item Response Theory. Global Mental Health. 2020;7:e3.
61. Sousa VD, Rojjanasrirat W. Translation, adaptation and validation of instruments or scales for use in cross-cultural health care research: a clear and user-friendly guideline. J Eval Clin Pract. 2011;17(2):268-74.
62. Wells SY, Morland LA, Torres EM, Kloezeman K, Mackintosh MA, Aarons GA. The Development of a Brief Version of the Posttraumatic Cognitions Inventory (PTCI-9). Assessment. 2019;26(2):193-208.

For peer review only - http://bmjopen.bmj.com/site/about/guidelines.xhtml

63. Moullin JC, Dickson KS, Stadnick NA, Rabin B, Aarons GA. Systematic review of the Exploration, Preparation, Implementation, Sustainment (EPIS) framework. 2019;14(1):1.
64. Gilson L. Editorial: building trust and value in health systems in low- and middle-income countries. Soc Sci Med. 2005;61(7):1381-4.
65. Gilson L, Elloker S, Olckers P, Lehmann U. Advancing the application of systems thinking in health: South African examples of a leadership of sensemaking for primary health care. Health research policy and systems / BioMed Central. 2014;12:30.
66. Kelly P, Hegarty J, Barry J, Dyer KR, Horgan A. A systematic review of the relationship between staff perceptions of organizational readiness to change and the process of innovation adoption in substance misuse treatment programs. J Subst Abuse Treat. 2017;80:6-25.
67. Brooke-Sumner C, Petersen-Williams P, Kruger J, Mahomed H, Myers B. 'Doing more with less': a qualitative investigation of perceptions of South African health service managers on implementation of health innovations. Health policy and planning. 2019;34(2):132-40.
68. Cleary SM, Molyneux S, Gilson L. Resources, attitudes and culture: an understanding of the factors that influence the functioning of accountability mechanisms in primary health care settings. BMC health services research. 2013;13:320-.
69. Furr RM, Bacharach, V. R. Psychometrics: An Introduction. Sage; 2006. p. 191-235.
70. Maphumulo WT, Bhengu BR. Challenges of quality improvement in the healthcare of South Africa post-apartheid: A critical review. Curationis. 2019;42(1):e1-e9.

For peer review only

For peer review only - http://bmjopen.bmj.com/site/about/guidelines.xhtml

Supplementary Table 1: Items removed from TCU-ORC before survey use based on research team discussion (23 total)

Item removed from original scale	Reason
A5 increasing program participation by clients	With high workloads and staff shortages this is not part of the expected role
A9 improving cognitive focus of clients during group counseling	Group counselling not currently provided
A19 improving billing/financial/accounting procedures	Clinic services provided free of charge
A30 Current pressures to make program changes come from board members or overseers	Stakeholder group not influential in government run and funded health facilities
A31 Current pressures to make program changes come from community groups	Stakeholder group not influential in government run and funded health facilities
A32 Current pressures to make program changes come from funding agencies	Stakeholder group not influential in government run and funded health facilities
A33 Current pressures to make program changes come from accreditation or licensing authorities	Stakeholder group not influential in government run and funded health facilities
B6 Policies limit use of the Internet for work-related needs at your program.	Most clinic staff do not have internet access at work or use for work purposes
B7 Computer problems are usually repaired promptly at your program	Most clinic staff do not use computers in day to day work
B10 You have convenient access to e-mail at work	Most clinic staff to not use email for work purposes
B14 More computers are needed for staff in your program to use	Clinic staff not expected to have computers for work use
B15 Most client records for your program are computerized	Electronic records system not implemented
B21 You used the Internet at work recently to access drug treatment information.	Clinic staff do not use internet for work purposes
B23 You have easy access for using the Internet at work	Clinic staff not expected to have internet access for work purposes
B26 You have a computer to use in your personal office space at work	Clinic staff not expected to have computer for work purposes
B29 The budget in your program allows staff to attend professional training.	General staff do not have knowledge about budgets and allocations, in service training most common
B30 Staff in your program feel comfortable using computers	Clinic staff not expected to use computers for work purposes
C12 12-step programs (AA/NA) are recommended to many of your clients	12 step programmes not accessible in most settings
C21 Behavior modification (contingency management) is used with many of your clients	Behaviour modification not implemented – no substance abuse behaviour change in PHC
C22 You have the skills needed to conduct effective individual counseling.	General staff not expected to provide individual counselling, individual counselling not widely implemented
C26 Cognitive theory (RET, RBT) guides much of your counseling.	Counselling not implemented
C31 Pharmacotherapy and related medications are important for many of your clients	Staff see not only substance abuse or mental health clients – medication will be widely used for other conditions
D11 Counselors in your program are given broad authority in treating their clients	Counselling not implemented by nursing staff

For peer review only

BMJ Open

Adaptation of the Texas Christian University Organisational Readiness for Change Short Form (TCU-ORC-SF) for use in primary health facilities in South Africa

Journal:	*BMJ Open*
Manuscript ID	bmjopen-2020-047320.R1
Article Type:	Original research
Date Submitted by the Author:	03-Aug-2021
Complete List of Authors:	Brooke-Sumner, Carrie; Medical Research Council of South Africa, Alcohol Tobacco and Other Drug Research Unit;<br>Petersen-Williams, Petal; South African Medical Research Council, Alcohol, Tobacco and Other Drug Research Unit<br>Wagener, Emma; University of the Western Cape, Faculty of Community and Health Sciences<br>Sorsdahl, Katherine; University of Cape Town Faculty of Health Sciences,<br>Aarons, Gregory; University of California, San Diego, Psychiatry; University of California, San Diego<br>Myers, Bronwyn; South African Medical Research Council, Alcohol and Drug Abuse Research Unit
**Primary Subject Heading**:	Health services research
Secondary Subject Heading:	Mental health, Medical education and training, Health policy
Keywords:	PRIMARY CARE, Organisational development < HEALTH SERVICES ADMINISTRATION & MANAGEMENT, Change management < HEALTH SERVICES ADMINISTRATION & MANAGEMENT

SCHOLARONE™
Manuscripts

I, the Submitting Author has the right to grant and does grant on behalf of all authors of the Work (as defined in the below author licence), an exclusive licence and/or a non-exclusive licence for contributions from authors who are: i) UK Crown employees; ii) where BMJ has agreed a CC-BY licence shall apply, and/or iii) in accordance with the terms applicable for US Federal Government officers or employees acting as part of their official duties; on a worldwide, perpetual, irrevocable, royalty-free basis to BMJ Publishing Group Ltd ("BMJ") its licensees and where the relevant Journal is co-owned by BMJ to the co-owners of the Journal, to publish the Work in this journal and any other BMJ products and to exploit all rights, as set out in our licence.

The Submitting Author accepts and understands that any supply made under these terms is made by BMJ to the Submitting Author unless you are acting as an employee on behalf of your employer or a postgraduate student of an affiliated institution which is paying any applicable article publishing charge ("APC") for Open Access articles. Where the Submitting Author wishes to make the Work available on an Open Access basis (and intends to pay the relevant APC), the terms of reuse of such Open Access shall be governed by a Creative Commons licence – details of these licences and which Creative Commons licence will apply to this Work are set out in our licence referred to above.

Other than as permitted in any relevant BMJ Author's Self Archiving Policies, I confirm this Work has not been accepted for publication elsewhere, is not being considered for publication elsewhere and does not duplicate material already published. I confirm all authors consent to publication of this Work and authorise the granting of this licence.

For peer review only

Adaptation of the Texas Christian University Organisational Readiness for Change Short Form (TCU-ORC-SF) for use in primary health facilities in South Africa

Carrie Brooke-Sumner[1,2], Petal Petersen-Williams[1,3], Emma Wagener[4], Katherine Sorsdahl[2], Gregory A. Aarons[5,6], Bronwyn Myers[1,3]

[1] Alcohol, Tobacco and Other Drug Research Unit, South African Medical Research Council

[2] Alan J Flisher Centre for Public Mental Health, Department of Psychiatry and Mental Health, University of Cape Town, South Africa

[3] Division of Addiction Psychiatry, Department of Psychiatry and Mental Health, University of Cape Town, South Africa

[4] Faculty of Community and Health Sciences, University of the Western Cape, Cape Town, South Africa

[5] Department of Psychiatry, University of California, La Jolla, San Diego, CA, USA

[6] Child and Adolescent Services Research Center, San Diego, CA, USA

Corresponding Author: Carrie.Brooke-Sumner@mrc.ac.za

Abstract

Objectives: The Texas Christian University Organisational Readiness for Change Scale (TCU-ORC) assesses factors influencing adoption of evidence-based practices. It has not been validated in low- and middle-income countries (LMIC). This study assessed its psychometric properties in a South African setting with the aim of adapting it into a shorter measure.

Methods: This study was conducted in 24 South African primary health care clinics in the Western Cape Province. The TCU-ORC and two other measures, the Organisational Readiness to Change

For peer review only

For peer review only - http://bmjopen.bmj.com/site/about/guidelines.xhtml

Assessment (ORCA) and the Checklist for Assessing Readiness for Implementation (CARI) were administered. The questionnaire was re-administered after 2 weeks to obtain data on test-retest reliability. Three hundred and ninety-five surveys were completed: 281 participants completed the first survey, and 118 re-completed the assessments.

Results: We used exploratory factor analysis (EFA) to identify latent dimensions represented in the data. Cronbach's alpha for each subscale was assessed and we examined the extent to which the subscales and total scale scores for the first and retest surveys correlated. Convergent validity was assessed by the correlation coefficient between the TCU-ORC, ORCA and CARI total scale scores. EFA resulted in a three-factor solution. The three subscales proposed are Clinic Organisational Climate (8 items), Motivational Readiness for Change (13 items), and Individual Change Efficacy (5 items) (26 items total). Cronbach's alpha for each subscale was >.80. The overall shortened scale had a test-retest correlation of r = .80, p < 0.01, acceptable convergent validity with the ORCA scale (r = .56, p < .05), moderate convergence with the CARI (r = 39, p<.05) and strong correlation with the original scale (r = 0.79, p < .05).

Conclusions: This study presents the first psychometric data on the TCU-ORC from a LMIC. The proposed shortened tool may be more feasible for use in LMICs.

Trial registration: Not applicable.

Article summary – strengths and limitations

- This study presents a pragmatic tool for assessing organisational readiness for change in primary health facilities in South Africa.

For peer review only - http://bmjopen.bmj.com/site/about/guidelines.xhtml

- Factor structure and psychometric properties including temporal stability and convergent validity are presented indicating usefulness of this adapted tool in assessing readiness for change and enabling innovation adoption.
- Adaptation of this tool is a based on data collected from staff in primary health facilities in South Africa indicating its potential for other nurse-led and managed primary health settings.
- Data collected reflect the sociodemographic and organisational context of health facilities and contextual adaptation may be required for other settings.

Keywords

Health system resilience, organisational readiness for change, factor analysis, innovation adoption, evidence-based practice, task-sharing, primary care

Background

The treatment gap for mental disorders in South Africa, like other low- and middle-income countries (LMIC) and high-income countries (HIC), is well documented, with increasing attention being directed towards addressing this gap by providing mental health services in primary care. With the reality of constrained budgets for health and human resource limitations in South Africa and globally, task-sharing of interventions to address mental disorders is gaining traction, whereby specific mental health services are delegated to non-specialist cadres with appropriate support and onward referral (1, 2). This approach is supported globally (3, 4) as a strategy for building health system resilience (5, 6) and is being investigated in a number of effectiveness and implementation studies in primary health care (PHC) settings in South Africa (2, 7-9). Little is known about the

For peer review only - http://bmjopen.bmj.com/site/about/guidelines.xhtml

readiness and capability of many primary health facilities to implement this innovation, and there is great variation in implementation factors across facilities. Previous work by our group has identified patterns of factors associated with capability for implementing mental health counselling at primary care facilities in the Western Cape province. These included good patient information systems, organized services and a strong management team (all supporting the provision of patient-centred cared). (10).The variability across facilities observed in this work indicates the need for feasible ways to assess readiness and identify barriers to implementation for these service innovations.

As the introduction of task-shared interventions can be complex, some interventions fail to be implemented despite a supportive policy environment (11). There may be several reasons for implementation failure in this context (12). For example, psychosocial interventions can be complex in nature (e.g. multiple sessions, psychological content); community contexts where patients live may challenge participation in sessions (e.g. poverty, violence) (13, 14); and importantly, the organisational setting of primary care facilities presents a complex environment for introducing mental health and other behavioural health innovations (e.g. heavy workloads, resource shortages, skills limitations, poor staff morale) (10, 15-21). Innovations need to be implemented by individuals embedded in complex organisations and difficult social contexts (22-24). This may help explain why many effective interventions do not transition to sustained routine practice in primary health organisations (25, 26), and why reducing the evidence-to-practice gap remains a priority for health services research.

Organisational readiness for change (ORC) provides a theoretical framework for conceptualising factors supporting or hindering initial adoption of new evidence-based practices. ORC, as defined

For peer review only - http://bmjopen.bmj.com/site/about/guidelines.xhtml

For peer review only

by Weiner, is a shared psychological state that relies on organisation members' motivation to change (change commitment) and belief in their own capacity to change (change efficacy) (27). Commonly identified components contributing to this shared state include organisational dynamics, climate and culture (particularly leadership from management), and individual members' characteristics and readiness to accept change, including attitudes to evidence based practices (27-31). Assessing ORC enables implementers to identify and develop approaches to reduce factors hindering innovation adoption and strengthen those factors that support adoption. In South Africa, constraints to innovation adoption include lack of resources, high workloads, inadequate staff skills, high levels of staff turnover, and complex interpersonal and hierarchical relationships (14, 19, 32, 33). The assessment of ORC in South African settings has substantial potential to enable identification and mitigation of these constraints.

A variety of instruments for assessing ORC have been developed (34) and used in HIC. Assessment of ORC in LMICs is limited to date but may have important potential for improving implementation of quality health care. The resources, operations, and outer system and inner organizational contexts and processes are likely to differ between LMICs and HICs. This study aimed to address this research gap through contextually adapting an instrument for assessing ORC in primary health settings in South Africa (35). The Texas Christian University ORC (TCU-ORC) scale (36) is among the most widely used of the ORC scales and has been tailored for use in health services research and with adaptation may be suitable for use in LMICs. Although there is good evidence for the instrument's validity in other settings (34), and it has been used in South Africa (37), little is known about the psychometric properties in LMICs and the South African context in particular. The TCU-ORC is a comprehensive measure of ORC assessing both individual

For peer review only - http://bmjopen.bmj.com/site/about/guidelines.xhtml

psychological and organizational and health system influences on ORC. However, with 125-items, its length may make it unpractical for routine use in busy and under-resourced services. There are continuing calls for development and testing of brief and pragmatic measures (e.g., low burden, important to stakeholders, actionable, psychometrically strong, related to theory) and such measures are more likely to be used in research and practice (38, 39).

This study was nested within a study known as Project MIND, a cluster randomised controlled trial evaluating two approaches to resourcing the integration of task-shared counselling for patients with depression and/or hazardous or harmful alcohol use (2). The Project MIND counselling programme comprises three-sessions of motivational interviewing and problem-solving therapy delivered by trained lay counsellors (2, 40). The integration of this service constitutes a change to the basket of health services currently available within primary care clinics, providing an opportunity for contextually validating the TCU-ORC. This study aimed to assess the psychometric properties of the TCU-ORC in South African primary care clinics and reduce the number of items to a smaller, more manageable and relevant tool for this setting.

Methods

Methods for the Project MIND trial (2) and this adaptation sub-study (35) are fully described elsewhere. Briefly, this study involved administering a set of ORC assessments to providers within Project MIND trial sites at two time points with the purpose of establishing the internal consistency, temporal stability and construct validity of the long-form TCU-ORC questionnaire.

Participants and Procedures

For peer review only - http://bmjopen.bmj.com/site/about/guidelines.xhtml

Data collection took place from July to December 2018 at the 24 facilities that participated in the MIND trial. Following a workshop that introduced the study in the facilities, health service employees (managers and staff) were requested to complete a paper-based, English language self-report survey, after granting written informed consent. English is the official language of business of the South African health system.

Participants at workshops included facility managers, operational managers, medical officers, psychiatric nurses, general nursing staff from the chronic disease care team, and lay counsellors. We aimed to secure participation of 12 staff members from each facility (288 participants in total), however the number of participants per workshop was at the discretion of the facility manager based on staff availability. Between one and two weeks after the workshop, Project MIND fieldworkers asked a sub-sample of participants (convenience sample) from each facility to re-complete the paper-based survey comprising the ORC measures to enable assessment of test-retest reliability. These fieldworkers also approached nursing staff directly to fill in the questionnaire to gain enough responses for the first survey. In total, 395 surveys were completed: 281 participants completed the first survey and 118 participants completed both the first and test-retest survey.

Measures

The survey comprised three measures of ORC; the TCU-ORC (36), the Organisational Readiness to Change Assessment (ORCA) (41) and the Checklist for Assessing Readiness for Implementation (CARI) (42).

For peer review only - http://bmjopen.bmj.com/site/about/guidelines.xhtml

The four domains of the TCU-ORC (125 items total) measure Motivational Readiness for Change (Subscale A: covering staff's readiness, efficacy and support needs for implementing new practices; 33 items; e.g. clinical staff at your program needs guidance in matching client needs with services); Institutional Resources (Subscale B: adequacy of infrastructure, training and management; 31 items; e.g. much time and attention are given to staff supervision when needed); Staff Attributes (Subscale C: staff skills, management planning and leadership; 31 items; e.g. learning and using new procedures are easy for you) and Organisational Climate (Subscale D: shared goals, communication structure, work environment; 30 items; e.g. the heavy staff workload reduces the effectiveness of your program). Items are scored on a five-point Likert scale ranging from strongly disagree to strongly agree. In development, these subscales showed acceptable reliability (alpha > .70 Subscale A; alpha > .60 Subscale B; alpha > .50 Subscale C; alpha > .50 Subscale D) (36).

The Organizational Readiness For Change Assessment (ORCA) (41) comprises 50 items across three scales. These assess (1) strength of evidence for the change to be introduced (9 items; e.g. the proposed change to the service delivery of the facility is supported by clinical experience with patients in other health care systems); (2) quality of the organisational context (22 items; e.g. in general in my facility, when there is agreement that change needs to happen we have the necessary support in terms of budget or financial resources) and (3) capacity for organisational facilitation of the change (19 items; e.g. communication will be maintained through regular meetings covering the intervention). Items are scored on a five-point Likert scale ranging from strongly disagree to strongly agree. There is evidence for the overall reliability and factor structure of the ORCA (alpha .74, .85 and .95 for the subscales 1, 2 and 3, respectively) (41).

For peer review only - http://bmjopen.bmj.com/site/about/guidelines.xhtml

The CARI checklist (42) includes 36 items that assess: (1) organisational capacity (five items; e.g. to what extent do you think technical assistance is available for the intervention being implemented); (2) organisational capacity (four items; e.g. to what extent do you think financial resources are adequate and available to introduce and sustain the intervention); (3) functional considerations (four items, e.g. to what extent do you think there is a system in place to share patient outcomes with staff and management), (4) organisational culture/climate (five items, e.g. to what extent do you think there are open lines of communication in place in this facility; (5) senior leadership (five items; e.g. to what extent do you think senior leadership is convinced of the value of this intervention; (6) staff capacity (five items; e.g. to what extent do you think staff show they are ready for this type of practice change); (7) implementation plan (three items; e.g. to what extent do you think there is intent to monitor fidelity of the intervention and (8) training; (five items, e.g. to what extent do you think adequate time will be set aside for training, reflection and practice). This instrument was selected because it contains items that are broadly similar in content and wording to the TCU-ORC items although data on its psychometric properties remain to be published. Neither of the additional instruments have been validated in LMIC to our knowledge.

Twenty-three items across the four subscales of the TCU-ORC were removed prior to the assessment as they were not appropriate for the South African PHC context, (See Supplementary Table 1 for item removal based on discussion with the research group), leaving 104 items in the ORC for further assessment. Some of the removed items related to computer availability. While staff in primary care settings are meant to have access to computers and the internet, in practice this is often not the case with staff having limited access to computers where these are available. Minor editing of items was carried out to improve readability and appropriateness for clinic staff (for example ‘your program’ was edited to read ‘your facility’).

For peer review only - http://bmjopen.bmj.com/site/about/guidelines.xhtml

Analyses

Stata 16 statistical software was used for analysis. Cases with >50% missing data were removed (43). Normality tests were conducted for all measures using the Shapiro-Wilks test in combination with visual inspection of the normality plots. We used exploratory factor analysis, specifically Principal Axis Factoring (PAF) to identify latent dimensions represented in the variables (44, 45). The factor solution was rotated using Oblique Oblimin rotation, which is suited when factors are possibly related, but measure different aspects of a construct (46). Factor extraction was determined on the basis of the theoretical structure of the TCU-ORC (36), as well as the Kaiser K0 rule for common factors, in combination with Horn's parallel analysis (47-49). Decisions around item retention were guided by item communalities and loading of items onto factors. Items with communalities of at least .70 are considered highly desirable (50). Although items with loadings greater than .40 are often retained (51), the decision was made to include items with factor loadings above .60 for parsimony due to the large number of items and to allow for a more stable factor solution.

Next, internal consistency of each subscale was assessed by calculating Cronbach's alpha of the reduced item subscales. Internal consistency is considered acceptable at values above .70 for generalisability (52). To assess temporal stability, we examined the extent to which the subscales and total scales for the first and retest surveys correlated with each other, with a correlation coefficient above .70 considered adequate. Convergent validity was assessed by calculating the correlation coefficient between the TCU-ORC, ORCA and CARI measures for the total scales (36, 41, 42).

Results

For peer review only - http://bmjopen.bmj.com/site/about/guidelines.xhtml

After removal of cases with less than 50% data, a total 275 participants with adequate responses who had completed the first survey and 118 participants who had completed both the first and retest survey were included in the analyses. Participants' sociodemographic characteristics are summarized in Table 1. The sample comprised predominantly female nursing staff, reflecting the staffing profile of primary clinics in South Africa. A large proportion had worked at their current facility for more than five years and the majority had been in their profession for more than five years (Table 1).

Exploratory factor analysis

Assumption testing for factor analysis

Normality of the TCU-ORC was established by a non-significant Shapiro-Wilk result (p = .11) and visual inspection of the Histogram and the Q-Q plot. Bartlett's test of sphericity indicated that correlations between items were sufficiently large for factor analysis ($\chi^2(5356) = 12099.13$, p < .01). The Kaiser-Meyer-Olkin (KMO) measure of sampling adequacy was found to be acceptable with a value of .70 (53).

Factor extraction. The initial extraction was run with the Kaiser rule specified, retaining factors with eigenvalues greater than 0. This identified 74 factors. A parallel analysis (PA) was run in Stata, using Glorfeld's (1995) extension and using the 99th percentile to compare eigenvalues in order to increase sensitivity (49).The 99th percentile analysis resulted in 15 factors.

When conducting a PA, factors with a large number of variables are prone to inaccuracies due to poorly defined factors (54). As this measure contains a large number of variables, it is likely that the results of the PA over extracted factors. The decision was made to specify four factors, according to these results and the theoretical basis of the original TCU-ORC (36). After the initial

For peer review only - http://bmjopen.bmj.com/site/about/guidelines.xhtml

extraction of four factors, we observed that multiple items from two subscales loaded onto one factor. Visual inspection of the scree plot confirmed three distinct points above the point of inflection (55), suggesting a three-factor solution. Subsequent analyses were based on the specification of a three-factor solution.

Factor loadings of the final factor structure are presented in Table 2. Eleven extractions were performed overall to reach the final three factor solution. This explained 72.4% of shared variance. We removed items with cross-loadings of .3 or above on any item and items were removed one at a time in sequential factor analysis runs (51). Sampling adequacy using the KMO Measure was assessed for each item as a post-estimation after each extraction. In each extraction, items with loadings less than .60 were removed after assessing how removing items affected the Cronbach's alpha for each subscale and whether internal consistency improved. The final three factor solution consists of 26 items, having removed 99 items and therefore providing a simplified, shortened version of the scale (Table 3).

Factor 1 'Clinic Organisational Climate' consists of 13 items, with loadings between .61 and .72. This factor is made up of items from the original Institutional Resources, Staff Attributes and Organizational Climate subscales. The revised subscale had a Cronbach's alpha of .90 and correlated with the original 'Organizational Climate' subscale (r = .82, p < .05).

Factor 2 'Motivational Readiness for Change' consists of eight items, with loadings between .64 and .78. The items in this subscale were all originally in the TCU-ORC's Motivational Readiness for Change subscale. This subscale had good reliability with a Cronbach's alpha of .93 and correlated with the original 'Motivational Readiness for Change' subscale (α = .79).

For peer review only - http://bmjopen.bmj.com/site/about/guidelines.xhtml

Factor 3 'Individual Change Efficacy' consists of five items, with factor loadings between .62 and .76. The items in this subscale all originated from the 'Staff Attributes' subscale. This subscale had a Cronbach's alpha of .83 and correlated with the original 'Individual Change Efficacy' subscale (α =.57).

Overall, the 26-item version of the TCU-ORC was strongly correlated with the original scale (r = .79, $p < .05$).

Temporal stability

A test-retest correlation coefficient between the initial and follow-up versions of the TCU-ORC was conducted, using only those items retained after PAF for both administration versions. Temporal stability was assessed for the overall scale as well as for each sub-scale. The correlation results are presented in Table 4. Subscales show adequate temporal stability for Clinic Organisational Climate ($r = .87$, $p < .01$), Motivational Readiness for Change ($r = .60$, $p < .01$), with exception of the Individual Change Efficacy subscale ($r = .53$, $p < .01$), Overall, the shortened scale was found to have good consistency across time with a test-retest correlation of $r = .80$, $p < .01$.

Convergent validity

The reliability coefficients of criterion measures were good for the ORCA (α = .96) and CARI (α = .96), supporting the interpretation of convergent correlations. The shortened version of the TCU-ORC was found to have acceptable convergent validity with the ORCA scale ($r = .56$, $p < .05$), with a moderate correlation with the CARI ($r = .39$, p < .05). Convergent validities of $r = .70$ are considered ideal, with validities less than $r = .50$ considered insufficient in organisational research (56) (54).

For peer review only - http://bmjopen.bmj.com/site/about/guidelines.xhtml

Discussion

Validated measures for assessing organizational functioning and dynamics in health organisations in LMICs are currently lacking (57). These measures are needed to assist health systems as they grapple with the need to be resilient and responsive to the changing health needs of the population (6, 32). To be applicable and useful, measures must be pragmatic and useful to the service providers involved in delivering and managing systems and facilities (38, 39). This study presents findings on the psychometric properties of the TCU-ORC amongst a sample of health workers from primary care settings in the Western Cape Province of South Africa. The sample was drawn from front-line providers in health facilities, who are key to influencing implementation of evidence-based practices through their role as 'street level bureaucrats' (58) who through their work have influence on the services received by facility clients. While the usefulness of this measure has been investigated by studies from a number of HIC (e.g. (59, 60)), this is the first investigation of its kind in primary care in South Africa and to our knowledge, in other LMICs.

The overall goal of this work was to develop a contextually relevant measure, feasible for use in primary care within South Africa (35). While the original measure has been widely used in better resourced settings, 125 items is not a feasible for use as a routine assessment in the busy and under-resourced South African health system. A shorter, pragmatic instrument capable of collecting essential information to inform implementation is required for completion by primary care staff with high workloads and multiple competing priorities (14). The need for reducing response burden and respondent fatigue is aligned with current thinking among global mental health researchers who have noted response burden as a threat to the quality of data collected by lengthy measures (61). The current study resulted in the reduction of the TCU-ORC to 26 items.

For peer review only - http://bmjopen.bmj.com/site/about/guidelines.xhtml

While this is a considerable reduction, further shortening may be needed to produce a scale truly feasible for routine use in primary care in other settings. Experience from HIC settings underscores the importance of ensuring implementation support measures are responsive to stakeholders' needs, present a low burden and have wide application across a multitude of settings (38, 39, 62). Scales with large number of items have been shown to be feasibly shortened to as low as 3 items per subscale retaining high degree of validity and reliability (alpha > .9) e.g. (63). With this in mind, we plan to conduct a Delphi study with stakeholders and experts from the South African primary care arena to further shorten the tool and enable integration of stakeholder perspectives. Subsequently a confirmatory factor analysis (CFA) will be conducted.

Factor analysis showed overlap between three of the original domains, suggesting a three-factor structure for the South African data, in contrast to the original four domains [(Motivational Readiness for Change (A), Institutional Resources (B) Staff Attributes (C) and Organisational Climate (D)]. The proposed domains for the South African data, as shown in Table 2 are (1) Clinic Organisational Climate, (13 items) (2) Pressures for Change (8 items) and (3) Individual change efficacy (5 items). Each domain and the overall scale showed strong internal consistency, adequate temporal stability, and moderate convergent validity with the ORCA and CARI measures. The temporal stability for the Individual Change Efficacy scale may reflect the unstable and uncertain climate within which health providers often operate. At the time of this study, many of the clinics were affected by community violence and service delivery protests that sometimes led to clinic closures or restricted service delivery. It is plausible that these contextual factors may have impacted on individual providers perceptions of change efficacy, especially as efficacy for change is often context dependent. However, more research is needed to identify and address such

For peer review only - http://bmjopen.bmj.com/site/about/guidelines.xhtml

"bridging factors" that demonstrate bi-directional influences of outer system and inner organizational contexts (64).

Three items with cross loadings >.3 (.3 to .5) were removed suggesting retained items were representing relatively unique constructs that were understood by respondents (57). The domain 'Clinic Organisational Climate' incorporates items from the Institutional Resources, Staff Attributes and Organisational Climates domains of the original TCU-ORC. This may be because institutional resources, staff attributes, and trusting supervisory relationships become essential prerequisites for staff's functioning within constrained environments. These are perceived as key aspects of the organizational climate and have been previously shown to group as elements of organisational functioning (10). Thirteen items were retained in this domain suggesting these are key to assessing organizational climate in this setting. Items included those relating to management, supervision, teamwork, leadership and trusting relationships, which aligns with other work investigating primary care functioning in South Africa (19, 32, 65, 66). Findings from qualitative work assessing primary care facilities' preparedness for mental health counselling implementation also suggest intersecting relationships between resource availability, management style and facility environment (21). The Pressures for Change domain remained largely consistent with the corresponding domain of the original TCU-ORC. Only a small number of items were retained from the C domain on Staff Attributes - these related to individual agency for change. Organisational theory outlines how individuals within an organisation can be resistant to change, particularly in the context of heavy workloads (27, 67) known to be present in South African facilities. South African facility managers are often clinicians, without management training, who work in high workload environments and indicate that workload constrains innovation adoption (68). The South African health system has a strong hierarchy of downward supervision and

For peer review only

For peer review only - http://bmjopen.bmj.com/site/about/guidelines.xhtml

management, compliance and targets and this may hinder frontline managers and staff from being responsive to patient needs and public health burden (69). At the same time, nurses are the majority of clinic staff and are key to organisational climate and change. The items retained in this scale may therefore be most relevant for assessing individual perceptions amongst nursing staff on their agency and willingness to change.

In relation to convergent validity, correlation of TCU-ORC-SF with the ORCA showed large effect, but with the CARI correlation showed medium effect. These two additional instruments were also designed to assess organisational readiness for change in HICs, and although some aspects of organisational culture, dynamics and functioning are translatable to other settings, others may not be robustly relevant in the South African PHC context. With regards to correlation of the shortened tool with the CARI, it is likely that the restricted range of responses in the CARI resulted in a lower correlation (70). The CARI items are measured on a 4-point scale, while the TCU-ORC items are measured on a 5-point scale. Nonetheless, it is the opinion of the researcher/s that these validity correlations between the ORCA, CARI and TCU-ORC scales are acceptably large to assume convergence. We propose that the revised domains presented here more closely capture the essentials of organisational readiness for change in South African PHC settings than the original measures. This adapted measure may enable progress on assessing readiness for implementation of evidence-based practices and addressing barriers in South African health facilities, identified as a prerequisite for implementation in our previous work (10). Further, there will be a wide variety of factors influencing readiness for change, particularly in different settings in LMIC and elsewhere, and detailed implementation research elucidating these elements would support the use or adaptation of this tool in different settings. The current work provides a base for

For peer review only

driving policy implementation to enable adoption of innovations to improve public health through strengthening primary healthcare platforms.

Limitations

Data for this study were collected from primary care facilities from the Western Cape province. This province is more well-resourced than other provinces. Data from the study showed a sociodemographic profile of facility staff that reflects the qualifications, professions, and work experience common across primary care settings in the country. This suggests appropriateness for the broader South African context, although there are important disparities in human and other resources and variations in burden of disease and contexts across provinces (5, 71) and additional adaptations could be required in other areasThe survey was in English which is not the first language of health providers in some settings, however English is the official business language of government agencies and health providers would be expected to be proficient. The ORCA and CARI measures have also not been adapted for use in LMIC use so there may be limitations in their validity for this context which may have influenced convergent validity findings.

Conclusion

This study has presented the contextual adaptation of the TCU-ORC in a LMIC setting. Based on the findings from this study, we propose a short form of the TCU-ORC, comprising 26 of 125 items. The three subscales proposed: Clinic Organisational Climate, Motivational Readiness for Change, and Individual Change Efficacy contain key items for assessing ORC in this context. This scale may be useful as a tool for health service managers to highlight potential roadblocks to the introduction of innovations that may need to be addressed when developing an implementation strategy. This shortened tool however may still be too lengthy for widespread practical use in

For peer review only - http://bmjopen.bmj.com/site/about/guidelines.xhtml

primary care services and further shortening involving stakeholders who will use the tool in practice is required.

Declarations

Ethics approval and consent to participate

Ethical approval was granted for this study by the Human Research Ethics Committee of the South African Medical Research Council (EC004-2-2015) and approval for working in facilities was also granted by the Western Cape Provincial Department of Health.

Consent for publication

Not applicable.

Patient and public involvement

It was not appropriate to involve patients or the public in this study since it focused on primary health staff as employees of the health system. We will present the shortened tool to staff and managers of the study facilities as part of the post-trial engagement meetings for Project MIND.

Availability of data and materials

Data from the study may be made available upon reasonable request.

Competing interests

The authors declare that they have no competing interests.

Funding statement

For peer review only

This study is funded jointly by the British Medical Research Council, Wellcome Trust, Department for International Development, the Economic and Social Research Council, the Global Challenges Research Fund (MR/M014290/1) as well as funding from the South African Medical Research Council (SAMRC, contract). CBS was supported as a postdoctoral fellow by funding from the SAMRC through its Division of Research Capacity Development under the Intra-Mural Postdoctoral Fellowship Programme from funding received from the South African National Treasury. The content hereof is the sole responsibility of the authors and does not necessarily represent the official views of the SAMRC or the funders.

Author contributions

CBS and BM conceptualised the study. CBS and PPW managed data collection. BM and KS are Principal Investigators on the Project MIND trial in which the study is nested. CBS and EW conducted factor analysis. EW provided statistical technical input and drafted some sections of the results. GA provided technical guidance on methods and analysis. All authors reviewed and contributed to the final version of the manuscript.

Acknowledgements

The authors thank Lesley-Anne Erasmus-Claasen, Yuche Jacobs and Nombuso Moshiga for their fieldwork on the study as well as all participating facilities, managers and staff.

For peer review only

For peer review only - http://bmjopen.bmj.com/site/about/guidelines.xhtml

Table 1 Sociodemographic data from PHC facility-based staff

Item	Category	Frequency	Percentage
Gender	Female	214	88.4%
	Male	28	11.6%
Age	20-30	14	5.3%
	31-40	72	27.2%
	41-50	73	27.5%
	51-60	84	31.7%
	61-65	22	8.3%
Ethnicity	Black African	136	50.2%
	Coloured	117	43.2%
	White	13	4.8%
	Indian/Asian	3	1.1%
	Other	2	0.7%
Time at facility	0-6 months	22	8.1%
	6-11 months	20	7.4%
	1-3 years	61	22.5%
	3-5 years	48	17.7%
	More than 5 years	120	44.3%
Education level	Completed high school	62	23.0%
	Some tertiary education	65	24.1%
	Bachelor's degree	51	18.9%
	Master's degree	7	2.6%
	Doctoral degree	3	1.1%
	Diploma in healthcare	74	27.3%
	Other	8	3.0%
Profession*	Medicine	25	9.1%
	Social work	6	2.1%
	Counselling	62	22.7%
	Psychology	2	0.7%
	Nursing	149	54.8%
	Administration	30	11.0%
	Other	18	6.6%
Time in profession	0-6 months	7	2.6%
	6-11 months	7	2.6%
	1-3 years	36	13.4%
	3-5 years	25	9.4%
	More than 5 years	192	72.0%

*Some participants selected working in more than one profession

For peer review only

For peer review only - http://bmjopen.bmj.com/site/about/guidelines.xhtml

Table 2 Principal axis factoring by domains of the original TCU-ORC

For peer review only

For peer review only - http://bmjopen.bmj.com/site/about/guidelines.xhtml

Subscale A: Motivational Readiness for Change (29 items included in factor analysis)<br>Subscale B: Institutional Resources (22 items included in factor analysis)<br>Subscale C: Staff Attributes (24 items included in factor analysis)<br>Subscale D: Organisational Climate (29 items included in factor analysis)													
Initial Factor 1		Final factor 1<br>Clinic Organisational Climate		Initial Factor 2		Final factor 2<br>Motivational Readiness for Change		Initial Factor 3		Final Factor 3<br>Individual Change Efficacy		Initial Factor 4	
Items	Loadings	Items	Loadings	Items	Loadings	Items	Loadings	Items	Loadings	Items	Loadings	Items	Loadings
A26	.32	B1	.66	A1	.56	A1	.68	B20	.35	C8	.68	B2	.38
B1	.64	B3	.61	A2	.51	A3	.77	C1	.44	C9	.71	C24	.41
B3	.53	B10	.68	A3	.55	A4	.78	C2	.30	C10	.76	D5	.36
B4	.39	B16	.71	A4	.59	A5	.77	C4	.48	C11	.62	D12	.35
B5	.56	B19	.65	A5	.63	A6	.73	C8	.50	C18	.62	D15	.34
B6	.51	C7	.66	A6	.59	A7	.70	C9	.61			D16	.34
B7	.60	D7	.62	A7	.57	A8	.70	C10	.52			D19	.44
B8	.59	D11	.65	A8	.58	A10	.64	C11	.47			D22	.54
B10	.69	D14	.66	A9	.61			C12	.34			D27	.33
B11	.64	D21	.70	A10	.69			C13	.31				
B12	.39	D25	.72	A11	.63			C14	.35				
B13	.44	D26	.66	A12	.64			C17	.44				
B14	.48	D28	.64	A13	.56			C18	.53				
B15	.39	D29	.61	A14	.55			C23	.45				
B16	.70			A15	.60			D6	.33				

For peer review only - http://bmjopen.bmj.com/site/about/guidelines.xhtml

B17	.58	A16	.59
B18	.52	A18	.30
B19	.62	A19	.50
B21	.45	A20	.46
C3	.44	A21	.49
C5	.34	A22	.37
C6	.53	A23	.51
C7	.65	A24	.34
C10	.33	A28	.38
C13	.32	A29	.30
C14	.49	C2	.31
C15	.35	C8	.36
C20	.38	C10	.34
C21	.41	C16	.40
C23	.39	C17	.32
D3	.42	C18	.43
D4	.53	D6	.45
D5	.62	D15	.32
D7	.61	D24	.36
D8	.67		
D9	.64		
D11	.60		
D13	.56		
D14	.67		

For peer review only

D16	.40
D17	.50
D18	.55
D20	.45
D21	.67
D23	.45
D25	.70
D26	.63
D28	.61
D29	.61

Extraction Method: Factor Analysis.
Rotation Method: Oblique Oblimin

For peer review only

Table 3 Excluded items and proposed revised domains*

Original TCU ORC domain	Number of items in initial factor analysis<br><br>(104 total)	Excluded items<br><br>(loadings < .6 through 11 extractions)	Proposed revised domains	Number of items in proposed scale
A Motivational Readiness for Change	29	Your facility needs guidance in<br><br>A2 using patient assessments to guide<br><br>clinical care<br><br>A9 defining its mission<br><br>A11 assigning or clarifying staff roles<br><br>A12 setting out accurate job descriptions for staff<br><br>A13 evaluating staff performance<br><br>A14 improving relations among staff<br><br>A15 improving communications among staff<br><br>A16 improving record keeping and information systems<br><br>A17 basic computer skills/programs<br><br>A18 specialized computer applications<br><br>A19 new developments and practices in your area of responsibility<br><br>A20 new procedures being used or planned<br><br>A21 continuous professional development<br><br>A22 new laws or regulations you need to know about<br><br>A23 management or supervisory responsibilities<br><br>Current pressures to make facility changes come from<br><br>A24 community members (patients)<br><br>A25 other staff members<br><br>A26 facility supervisors or managers<br><br>A27 district level managers<br><br>A28 community groups<br><br>A29 national and provincial DOH structures	**Motivational Readiness for Change**<br><br>Clinical staff at your facility needs guidance in:<br><br>A1 assessing patient needs<br><br>A3 using patient assessments to document patient improvements<br><br>A4 matching patient needs with services<br><br>A5 improving relationship with patients<br><br>A6 improving patient thinking and problem-solving skills to manage chronic disease<br><br>A7 improving behavioural management of patients<br><br>A8 identifying and using evidence-based practices<br><br>Your facility needs guidance in:<br><br>A10 setting specific goals for improving services	8

For peer review only

For peer review only - http://bmjopen.bmj.com/site/about/guidelines.xhtml

B Institutional Resources	22	B2 Frequent staff turnover is a problem for your facility<br><br>B4 The space in your facilities are adequate for conducting group or individual counselling<br><br>B5 You have clinical supervisors who are capable and qualified<br><br>B6 You learned new skills or techniques at a professional training in the past year<br><br>B7 Much time and attention are given to staff supervision when needed<br><br>B8 Staff in your facility are able to spend the time needed with patients<br><br>B9 Equipment at your facility is mostly old and outdated<br><br>B11 Support staff in your facility have the skills they need to do their jobs<br><br>B12 Offices in your facility allow the privacy needed for individual counselling<br><br>B13 Your facility holds regular in-service training<br><br>B14 Your facility has enough staff to meet current patient needs<br><br>B15 Clinical staff in your program are well-trained<br><br>B17 Offices and equipment in your facility are adequate<br><br>B18 Your facility provides a comfortable reception/waiting area for patients<br><br>B20 A larger support staff is needed to help meet needs at your program.<br><br>B21 Staff in your facility are able to attend professional training<br><br>B22 Staff concerns are ignored in most decisions made in your facility<br><br>D29 You feel encouraged to try new and different techniques	**Clinic organisational climate**<br><br>B1 You have good facility management at your facility<br><br>B3 Staff training and continuing education are priorities in your facility<br><br>B10 Clinical and management decisions for your facility are well planned<br><br>B16 You have confidence in how decisions at your facility are made<br><br>B19 Staff meet frequently with clinical supervisors about patient needs and progress<br><br>C7 Your facility encourages and supports professional growth<br><br>D7 Ideas and suggestions in your facility get fair consideration by management<br><br>D11 Your facility staff is always kept well informed<br><br>D14 Your facility operates with clear goals and objectives<br><br>D21 The formal and informal communication channels in your facility work very well<br><br>D25 Management fully trusts professional judgments of staff in your facility<br><br>D26 Staff members always feel free to ask questions and express concerns in your facility<br><br>D28 Management for your facility has a clear plan for its future	13
C Staff Attributes	24	C1 You have the skills needed to effectively perform your role<br><br>C2 Other staff often ask your advice<br><br>C3 You are satisfied with your present job	**Individual change efficacy**<br><br>C8 You are effective and confident in doing your job<br><br>C9 You are able to adapt quickly when you have to make changes	5

For peer review only - http://bmjopen.bmj.com/site/about/guidelines.xhtml

		C4 Learning and using new procedures are easy for you<br><br>C5 You are considered an experienced source of advice about services and/or facility management<br><br>C6 You feel appreciated for the job you do at work<br>C12 You regularly influence the decisions<br><br>of other staff you work with<br><br>C13 You usually accomplish whatever you set your mind on<br><br>C14 You do a good job of regularly updating<br><br>and improving your skills<br><br>C15 You regularly read professional articles<br><br>or books related to your work<br><br>C16 Other staff often ask for your opinions<br><br>about issues in the facility<br><br>C17 You are willing to try new ideas even if some staff members are reluctant<br><br>C19 You are sometimes too cautious or slow to make changes<br><br>C20 You are proud to tell others where you work<br><br>C21 You like the people you work with<br><br>C22 You are viewed as a leader by the staff you work with<br><br>C23 You consistently plan ahead and carry out your plans<br><br>C24 You would like to find a job somewhere else	C10 Keeping your professional skills up-to-date is a priority for you<br><br>C11 You give high value to the work you do<br><br>C18 You frequently share your knowledge of new ideas with others	
D Organisational Climate	29	D1 Some staff members seem confused about the main goals for your facility<br><br>D2 The heavy staff workload reduces the effectiveness of your facility<br><br>D3 You frequently hear good ideas from other staff for improving services<br><br>D4 Care plans and decisions for patients in your facility often get revised by a clinical supervisor<br><br>D5 The general attitude in your facility is to accept new and changing technology or procedures<br><br>D6 More open discussions about facility issues are needed where you work		

For peer review only - http://bmjopen.bmj.com/site/about/guidelines.xhtml

		D8 Staff members at your facility work together as a team<br><br>D9 Your duties are clearly related to the goals for your facility<br><br>D10 You are under too many pressures to do your job effectively<br><br>D12 New ways of working from staff are discouraged where you work<br><br>D13 Mutual trust and cooperation among staff in your program are strong<br><br>D15 Staff members at your facility often show signs of high stress and strain<br><br>D16 It is easy to change procedures at your facility to meet new conditions<br><br>D17 Staff in your facility can try out<br><br>different techniques to improve their effectiveness<br><br>D18 Staff members at your facility get along very well<br><br>D19 Staff members are given too many rules in your facility<br><br>D20 Staff members at your program are quick<br><br>to help one another when needed<br><br>D22 There is too much friction among staff members you work with<br><br>D23 Staff members at your facility understand the work of the facility fits as part of the health improvement in your community<br><br>D24 Some staff in your facility do not do their fair share of work<br><br>D27 Staff frustration is common where you work		

* 'A' items refer to the Motivational Readiness for Change scale, 'B' items refer to the Institutional Resources scale, 'C' items refer to the Staff Attributes scale, 'D' items refer to the Organisational Climate scale.

For peer review only - http://bmjopen.bmj.com/site/about/guidelines.xhtml

Table 4 Internal consistency and retest reliability of the new subscales

Subscale/ Factor	Description	Cronbach's Alpha	Temporal stability (*r*)
Clinic Organizational Climate	Management practices, trust, leadership, communication, appreciation of staff, facility resources, attitudes to change	.90	.87**
Motivational Readiness for Change	Areas of need for facility improvement, and staff development	.91	.60**
Individual Change Efficacy	Individual self-efficacy, adaptability, knowledge and skills	.83	.53**
Total revised scale		.86	.80**

** Significant at the .01 level

References

1. Department of Health RoSA. National Mental Health Policy Framework and Strategic Plan 2013-2020. Pretoria: Department of Health South Africa, 2013.
2. Myers B, Lund C, Lombard C, Joska J, Levitt N, Butler C, et al. Comparing dedicated and designated models of integrating mental health into chronic disease care: study protocol for a cluster randomized controlled trial. Trials. 2018;19(1):185.
3. De Silva MJ, Ryan G. Global mental health in 2015: 95% implementation. The lancet Psychiatry. 2016;3(1):15-7.
4. Ciapponi A, Lewin S, Herrera CA, Opiyo N, Pantoja T, Paulsen E, et al. Delivery arrangements for health systems in low-income countries: an overview of systematic reviews. Cochrane Database Syst Rev. 2017;9:Cd011083.
5. Fusheini A, Eyles J. Achieving universal health coverage in South Africa through a district health system approach: conflicting ideologies of health care provision. BMC health services research. 2016;16(1):558-.
6. Barasa EW, Cloete K, Gilson L. From bouncing back, to nurturing emergence: reframing the concept of resilience in health systems strengthening. Health policy and planning. 2017;32(suppl_3):iii91-iii4.
7. Lund C, Alem A, Schneider M, Hanlon C, Ahrens J, Bandawe C, et al. Generating evidence to narrow the treatment gap for mental disorders in sub-Saharan Africa: rationale, overview and methods of AFFIRM. Epidemiol Psychiatr Sci. 2015;24(3):233-40.

For peer review only - http://bmjopen.bmj.com/site/about/guidelines.xhtml

8. Fairall L, Petersen I, Zani B, Folb N, Georgeu-Pepper D, Selohilwe O, et al. Collaborative care for the detection and management of depression among adults receiving antiretroviral therapy in South Africa: study protocol for the CobALT randomised controlled trial. Trials. 2018;19(1):193.
9. Petersen I, Bhana A, Fairall LR, Selohilwe O, Kathree T, Baron EC, et al. Evaluation of a collaborative care model for integrated primary care of common mental disorders comorbid with chronic conditions in South Africa. BMC psychiatry. 2019;19(1):107.
10. Myers B, Breuer E, Lund C, Petersen Williams P, van der Westhuizen C, Brooke-Sumner C, et al. Assessing capability for implementing mental health counselling within primary care facilities in a middle-income country: A feasibility study. Journal of psychiatric and mental health nursing. 2019;26(5-6):163-74.
11. Hanson K, Cleary S, Schneider H, Tantivess S, Gilson L. Scaling up health policies and services in low- and middle-income settings. BMC health services research. 2010;10 Suppl 1:I1.
12. Jacobs SR, Weiner BJ, Reeve BB, Hofmann DA, Christian M, Weinberger M. Determining the predictors of innovation implementation in healthcare: a quantitative analysis of implementation effectiveness. BMC health services research. 2015;15:6.
13. Eyles J, Harris B, Fried J, Govender V, Munyewende P. Endurance, resistance and resilience in the South African health care system: case studies to demonstrate mechanisms of coping within a constrained system. BMC health services research. 2015;15(1):432.
14. Brooke-Sumner C, Petersen-Williams P, Kruger J, Mahomed H, Myers B. 'Doing more with less': a qualitative investigation of perceptions of South African health service managers on implementation of health innovations. Health policy and planning. 2019;34(2):132-40.
15. Lehmann U, Gilson L. Actor interfaces and practices of power in a community health worker programme: a South African study of unintended policy outcomes. Health policy and planning. 2013;28(4):358-66.
16. Gopinathan U, Lewin S, Glenton C. Implementing large-scale programmes to optimise the health workforce in low- and middle-income settings: a multicountry case study synthesis. Tropical medicine & international health : TM & IH. 2014;19(12):1437-56.
17. Innis J, Dryden-Palmer K, Perreira T, Berta W. How do health care organizations take on best practices? A scoping literature review. Int J Evid Based Healthc. 2015;13(4):254-72.
18. Freeman M. Global mental health in low and middle income, especially African countries. Epidemiol Psychiatr Sci. 2016;25(6):503-5.
19. Gilson L, Barasa E, Nxumalo N, Cleary S, Goudge J, Molyneux S, et al. Everyday resilience in district health systems: emerging insights from the front lines in Kenya and South Africa. BMJ global health. 2017;2(2):e000224.
20. Mbau R, Gilson L. Influence of organisational culture on the implementation of health sector reforms in low- and middle-income countries: a qualitative interpretive review. Global health action. 2018;11(1):1462579.
21. Oboirien K, Harris B, Goudge J, Eyles J. Implementation of district-based clinical specialist teams in South Africa: Analysing a new role in a transforming system. BMC health services research. 2018;18(1):600.
22. Lund C, Brooke-Sumner C, Baingana F, Baron EC, Breuer E, Chandra P, et al. Social determinants of mental disorders and the Sustainable Development Goals: a systematic review of reviews. The Lancet Psychiatry.5(4):357-69.
23. Aarons GA, Hurlburt M, Horwitz SM. Advancing a conceptual model of evidence-based practice implementation in public service sectors. Adm Policy Ment Hlth. 2011;38.
24. Cresswell K, Sheikh A. Organizational issues in the implementation and adoption of health information technology innovations: an interpretative review. Int J Med Inform. 2013;82(5):e73-86.

For peer review only - http://bmjopen.bmj.com/site/about/guidelines.xhtml

25. Lau R, Stevenson F, Ong BN, Dziedzic K, Treweek S, Eldridge S, et al. Achieving change in primary care--effectiveness of strategies for improving implementation of complex interventions: systematic review of reviews. BMJ open. 2015;5(12):e009993.
26. Lau R, Stevenson F, Ong BN, Dziedzic K, Treweek S, Eldridge S, et al. Achieving change in primary care--causes of the evidence to practice gap: systematic reviews of reviews. Implementation Science. 2016;11:1-39.
27. Weiner BJ. A theory of organizational readiness for change. Implementation Science: IS. 2009;4:67-.
28. Aarons GA. Measuring provider attitudes toward evidence-based practice: consideration of organizational context and individual differences. Child and adolescent psychiatric clinics of North America. 2005;14(2):255-71, viii.
29. Aarons GA, Cafri G, Lugo L, Sawitzky A. Expanding the domains of attitudes towards evidence-based practice: the evidence based practice attitude scale-50. Adm Policy Ment Health. 2012;39(5):331-40.
30. Attieh R, Gagnon MP, Estabrooks CA, Legare F, Ouimet M, Roch G, et al. Organizational readiness for knowledge translation in chronic care: a review of theoretical components. Implementation science : IS. 2013;8:138.
31. Williams B, Perillo S, Brown T. What are the factors of organisational culture in health care settings that act as barriers to the implementation of evidence-based practice? A scoping review. Nurse education today. 2015;35(2):e34-41.
32. Gilson L. Trust and the development of health care as a social institution. Soc Sci Med. 2003;56(7):1453-68.
33. Gilson L, Palmer N, Schneider H. Trust and health worker performance: exploring a conceptual framework using South African evidence. Social Science & Medicine (1982). 2005;61(7):1418-29.
34. Gagnon M-P, Attieh R, Ghandour EK, Légaré F, Ouimet M, Estabrooks CA, et al. A Systematic Review of Instruments to Assess Organizational Readiness for Knowledge Translation in Health Care. PloS one. 2014;9(12):e114338.
35. Brooke-Sumner C, Sorsdahl K, Lombard C, Petersen-Williams P, Myers B. Protocol for development and validation of a context-appropriate tool for assessing organisational readiness for change in primary health clinics in South Africa. BMJ open. 2018;8(4).
36. Lehman WE, Greener JM, Simpson DD. Assessing organizational readiness for change. Journal of substance abuse treatment. 2002;22(4):197-209.
37. Bowles S, Louw J, Myers B. Perceptions of organizational functioning in substance abuse treatment facilities in South Africa. International journal of mental health and addiction. 2011;9(3):308-19.
38. Glasgow RE, Riley WT. Pragmatic measures: what they are and why we need them. Am J Prev Med. 2013;45(2):237-43.
39. Glasgow RE. What does it mean to be pragmatic? Pragmatic methods, measures, and models to facilitate research translation. Health Educ Behav. 2013;40(3):257-65.
40. Sorsdahl K, Naledi T, Lund C, Levitt NS, Joska JA, Stein DJ, et al. Integration of mental health counselling into chronic disease services at the primary health care level: Formative research on dedicated versus designated strategies in the Western Cape, South Africa. 2020:1355819620954232.
41. Helfrich CD, Li YF, Sharp ND, Sales AE. Organizational readiness to change assessment (ORCA): development of an instrument based on the Promoting Action on Research in Health Services (PARIHS) framework. Implementation science : IS. 2009;4:38.
42. Barwick M. Checklist to assess organizational readines s (CARI) for EIP Implementation. Toronto, Ontario: Hospital for Sick Children: 2011.

For peer review only

For peer review only - http://bmjopen.bmj.com/site/about/guidelines.xhtml

43. Van den Broeck J, Argeseanu Cunningham S, Eeckels R, Herbst K. Data Cleaning: Detecting, Diagnosing, and Editing Data Abnormalities. PLOS Medicine. 2005;2(10):e267.
44. Henson R, Roberts J. Use of Exploratory Factor Analysis in Published ResearchCommon Errors and Some Comment on Improved Practice. Educational and Psychological Measurement - EDUC PSYCHOL MEAS. 2006;66:393-416.
45. Mooi E, Sarstedt, M., & Mooi-Reci, I. . Principal component and factor analysis. Market Research. Singapore: Springer; 2018. p. pp 265-311.
46. Fabrigar LR, Wegener DT, MacCallum RC, Strahan EJ. Evaluating the use of exploratory factor analysis in psychological research. Psychological Methods. 1999;4(3):272-99.
47. Ledesma RD, Valero-Mora, P. Determining the number of factors to retain in EFA: An easy-to-use computer program for carrying out parallel analysis. Practical assessment, research, and evaluation. 2007;12(1):2.
48. Hayton JC, Allen DG, Scarpello V. Factor Retention Decisions in Exploratory Factor Analysis: A Tutorial on Parallel Analysis. Organizational Research Methods. 2004;7(2):191-205.
49. Dinno A. Implementing Horn's Parallel Analysis for Principal Component Analysis and Factor Analysis. Stata Journal. 2009;9:291-8.
50. Field A. Disscovering statistics using IMB SPSS statistics: Thousand Oaks: SAGE Publications Inc; 2013.
51. Costello AB, Osborne J. Best Practices in Exploratory Factor Analysis: Four Recommendations for Getting the Most From Your Analysis. Practical Assessment, Research & Evaluation. 2005;10:1-9.
52. Bartholomew DJ, Knott, M., & Moustaki, I. Latent variable models and factor analysis: A unified approach: John Wiley & Sons.; 2011.
53. Pett MA, Lackey, N. R., & Sullivan, J. J. Making sense of factor analysis. Thousand Oaks: Sage Publications; 2003.
54. Jones JM. The Influence of a Proposed Margin Criterion on the Accuracy of Parallel Analysis in Conditions Engendering Underextraction Masters Theses & Specialist Projects: https://digitalcommons.wku.edu/theses/2446; 2018.

55. Cattell RB. The Scree Test For The Number Of Factors. Multivariate Behavioral Research. 1966;1(2):245-76.
56. Carlson KD, Herdman, A. O. Understanding the impact of convergent validity on research results. Organizational Research Methods. 2012;15(1):17-32.
57. Uy J, Macarayan EK, Ratcliffe HL, Miller K, Otupiri E, Awoonor-Williams JK, et al. Preliminary validation of the PRImary care facility Management Evaluation tool (PRIME-Tool), a national facility management survey implemented in Ghana. BMC health services research. 2019;19(1):937.
58. Walker L, Gilson L. 'We are bitter but we are satisfied': nurses as street-level bureaucrats in South Africa. Soc Sci Med. 2004;59(6):1251-61.
59. Rampazzo L, De Angeli M, Serpelloni G, Simpson DD, Flynn PM. Italian Survey of Organizational Functioning and Readiness for Change: A Cross-Cultural Transfer of Treatment Assessment Strategies. 12: Karger AG. p. 176-81.
60. Rowan-Szal GA, Greener JM, Joe GW, Simpson DD. Assessing program needs and planning change. Journal of substance abuse treatment. 2007;33(2):121-9.
61. Haroz EE, Kane JC, Nguyen AJ, Bass JK, Murray LK, Bolton P. When less is more: reducing redundancy in mental health and psychosocial instruments using Item Response Theory. Global Mental Health. 2020;7:e3.
62. Sousa VD, Rojjanasrirat W. Translation, adaptation and validation of instruments or scales for use in cross-cultural health care research: a clear and user-friendly guideline. J Eval Clin Pract. 2011;17(2):268-74.

For peer review only - http://bmjopen.bmj.com/site/about/guidelines.xhtml

63. Wells SY, Morland LA, Torres EM, Kloezeman K, Mackintosh MA, Aarons GA. The Development of a Brief Version of the Posttraumatic Cognitions Inventory (PTCI-9). Assessment. 2019;26(2):193-208.
64. Moullin JC, Dickson KS, Stadnick NA, Rabin B, Aarons GA. Systematic review of the Exploration, Preparation, Implementation, Sustainment (EPIS) framework. 2019;14(1):1.
65. Gilson L. Editorial: building trust and value in health systems in low- and middle-income countries. Soc Sci Med. 2005;61(7):1381-4.
66. Gilson L, Elloker S, Olckers P, Lehmann U. Advancing the application of systems thinking in health: South African examples of a leadership of sensemaking for primary health care. Health research policy and systems / BioMed Central. 2014;12:30.
67. Kelly P, Hegarty J, Barry J, Dyer KR, Horgan A. A systematic review of the relationship between staff perceptions of organizational readiness to change and the process of innovation adoption in substance misuse treatment programs. J Subst Abuse Treat. 2017;80:6-25.
68. Brooke-Sumner C, Petersen-Williams P, Kruger J, Mahomed H, Myers B. 'Doing more with less': a qualitative investigation of perceptions of South African health service managers on implementation of health innovations. Health policy and planning. 2019;34(2):132-40.
69. Cleary SM, Molyneux S, Gilson L. Resources, attitudes and culture: an understanding of the factors that influence the functioning of accountability mechanisms in primary health care settings. BMC health services research. 2013;13:320-.
70. Furr RM, Bacharach, V. R. Psychometrics: An Introduction. Sage; 2006. p. 191-235.
71. Maphumulo WT, Bhengu BR. Challenges of quality improvement in the healthcare of South Africa post-apartheid: A critical review. Curationis. 2019;42(1):e1-e9.

For peer review only

For peer review only - http://bmjopen.bmj.com/site/about/guidelines.xhtml

Supplementary Table 1: Items removed from TCU-ORC before survey use based on research team discussion (23 total)

Item removed from original scale	Reason
A5 increasing program participation by clients	With high workloads and staff shortages this is not part of the expected role
A9 improving cognitive focus of clients during group counseling	Group counselling not currently provided
A19 improving billing/financial/accounting procedures	Clinic services provided free of charge
A30 Current pressures to make program changes come from board members or overseers	Stakeholder group not influential in government run and funded health facilities
A31 Current pressures to make program changes come from community groups	Stakeholder group not influential in government run and funded health facilities
A32 Current pressures to make program changes come from funding agencies	Stakeholder group not influential in government run and funded health facilities
A33 Current pressures to make program changes come from accreditation or licensing authorities	Stakeholder group not influential in government run and funded health facilities
B6 Policies limit use of the Internet for work-related needs at your program.	Most clinic staff do not have internet access at work or use for work purposes
B7 Computer problems are usually repaired promptly at your program	Most clinic staff do not use computers in day to day work
B10 You have convenient access to e-mail at work	Most clinic staff to not use email for work purposes
B14 More computers are needed for staff in your program to use	Clinic staff not expected to have computers for work use
B15 Most client records for your program are computerized	Electronic records system not implemented
B21 You used the Internet at work recently to access drug treatment information.	Clinic staff do not use internet for work purposes
B23 You have easy access for using the Internet at work	Clinic staff not expected to have internet access for work purposes
B26 You have a computer to use in your personal office space at work	Clinic staff not expected to have computer for work purposes
B29 The budget in your program allows staff to attend professional training.	General staff do not have knowledge about budgets and allocations, in service training most common
B30 Staff in your program feel comfortable using computers	Clinic staff not expected to use computers for work purposes
C12 12-step programs (AA/NA) are recommended to many of your clients	12 step programmes not accessible in most settings
C21 Behavior modification (contingency management) is used with many of your clients	Behaviour modification not implemented – no substance abuse behaviour change in PHC
C22 You have the skills needed to conduct effective individual counseling.	General staff not expected to provide individual counselling, individual counselling not widely implemented
C26 Cognitive theory (RET, RBT) guides much of your counseling.	Counselling not implemented
C31 Pharmacotherapy and related medications are important for many of your clients	Staff see not only substance abuse or mental health clients – medication will be widely used for other conditions
D11 Counselors in your program are given broad authority in treating their clients	Counselling not implemented by nursing staff

For peer review only

BMJ Open

Adaptation of the Texas Christian University Organisational Readiness for Change Short Form (TCU-ORC-SF) for use in primary health facilities in South Africa

Journal:	*BMJ Open*
Manuscript ID	bmjopen-2020-047320.R2
Article Type:	Original research
Date Submitted by the Author:	05-Oct-2021
Complete List of Authors:	Brooke-Sumner, Carrie; Medical Research Council of South Africa, Alcohol Tobacco and Other Drug Research Unit; Petersen-Williams, Petal; South African Medical Research Council, Alcohol, Tobacco and Other Drug Research Unit Wagener, Emma; University of the Western Cape, Faculty of Community and Health Sciences Sorsdahl, Katherine; University of Cape Town Faculty of Health Sciences, Aarons, Gregory; University of California, San Diego, Psychiatry; University of California, San Diego Myers, Bronwyn; South African Medical Research Council, Alcohol and Drug Abuse Research Unit
**Primary Subject Heading**:	Health services research
Secondary Subject Heading:	Mental health, Medical education and training, Health policy
Keywords:	PRIMARY CARE, Organisational development < HEALTH SERVICES ADMINISTRATION & MANAGEMENT, Change management < HEALTH SERVICES ADMINISTRATION & MANAGEMENT

SCHOLARONE™
Manuscripts

I, the Submitting Author has the right to grant and does grant on behalf of all authors of the Work (as defined in the below author licence), an exclusive licence and/or a non-exclusive licence for contributions from authors who are: i) UK Crown employees; ii) where BMJ has agreed a CC-BY licence shall apply, and/or iii) in accordance with the terms applicable for US Federal Government officers or employees acting as part of their official duties; on a worldwide, perpetual, irrevocable, royalty-free basis to BMJ Publishing Group Ltd ("BMJ") its licensees and where the relevant Journal is co-owned by BMJ to the co-owners of the Journal, to publish the Work in this journal and any other BMJ products and to exploit all rights, as set out in our licence.

The Submitting Author accepts and understands that any supply made under these terms is made by BMJ to the Submitting Author unless you are acting as an employee on behalf of your employer or a postgraduate student of an affiliated institution which is paying any applicable article publishing charge ("APC") for Open Access articles. Where the Submitting Author wishes to make the Work available on an Open Access basis (and intends to pay the relevant APC), the terms of reuse of such Open Access shall be governed by a Creative Commons licence – details of these licences and which Creative Commons licence will apply to this Work are set out in our licence referred to above.

Other than as permitted in any relevant BMJ Author's Self Archiving Policies, I confirm this Work has not been accepted for publication elsewhere, is not being considered for publication elsewhere and does not duplicate material already published. I confirm all authors consent to publication of this Work and authorise the granting of this licence.

For peer review only

Adaptation of the Texas Christian University Organisational Readiness for Change Short Form (TCU-ORC-SF) for use in primary health facilities in South Africa

Carrie Brooke-Sumner[1,2], Petal Petersen-Williams[1,3], Emma Wagener[4], Katherine Sorsdahl[2], Gregory A. Aarons[5,6], Bronwyn Myers[1,3]

[1] Alcohol, Tobacco and Other Drug Research Unit, South African Medical Research Council

[2] Alan J Flisher Centre for Public Mental Health, Department of Psychiatry and Mental Health, University of Cape Town, South Africa

[3] Division of Addiction Psychiatry, Department of Psychiatry and Mental Health, University of Cape Town, South Africa

[4] Faculty of Community and Health Sciences, University of the Western Cape, Cape Town, South Africa

[5] Department of Psychiatry, University of California, La Jolla, San Diego, CA, USA

[6] Child and Adolescent Services Research Center, San Diego, CA, USA

Corresponding Author: Carrie.Brooke-Sumner@mrc.ac.za

Abstract

Objectives: The Texas Christian University Organisational Readiness for Change Scale (TCU-ORC) assesses factors influencing adoption of evidence-based practices. It has not been validated

For peer review only - http://bmjopen.bmj.com/site/about/guidelines.xhtml

in low- and middle-income countries (LMIC). This study assessed its psychometric properties in a South African setting with the aim of adapting it into a shorter measure.

Methods: This study was conducted in 24 South African primary health care clinics in the Western Cape Province. The TCU-ORC and two other measures, the Organisational Readiness to Change Assessment (ORCA) and the Checklist for Assessing Readiness for Implementation (CARI) were administered. The questionnaire was re-administered after 2 weeks to obtain data on test-retest reliability. Three hundred and ninety-five surveys were completed: 281 participants completed the first survey, and 118 re-completed the assessments.

Results: We used exploratory factor analysis (EFA) to identify latent dimensions represented in the data. Cronbach's alpha for each subscale was assessed and we examined the extent to which the subscales and total scale scores for the first and retest surveys correlated. Convergent validity was assessed by the correlation coefficient between the TCU-ORC, ORCA and CARI total scale scores. EFA resulted in a three-factor solution. The three subscales proposed are Clinic Organisational Climate (8 items), Motivational Readiness for Change (13 items), and Individual

For peer review only - http://bmjopen.bmj.com/site/about/guidelines.xhtml

Change Efficacy (5 items) (26 items total). Cronbach's alpha for each subscale was >.80. The overall shortened scale had a test-retest correlation of $r = .80$, $p < 0.01$, acceptable convergent validity with the ORCA scale ($r = .56$, $p < .05$), moderate convergence with the CARI ($r = 39$, $p<.05$) and strong correlation with the original scale ($r = 0.79$, $p < .05$).

Conclusions: This study presents the first psychometric data on the TCU-ORC from a LMIC. The proposed shortened tool may be more feasible for use in LMICs.

Trial registration: Not applicable.

Article summary – strengths and limitations

- This study presents a pragmatic tool for assessing organisational readiness for change in primary health facilities in South Africa.
- Factor structure and psychometric properties including temporal stability and convergent validity are presented indicating usefulness of this adapted tool in assessing readiness for change and enabling innovation adoption.

For peer review only

- Adaptation of this tool is a based on data collected from staff in primary health facilities in South Africa indicating its potential for other nurse-led and managed primary health settings.
- Data collected reflect the sociodemographic and organisational context of health facilities and contextual adaptation may be required for other settings.

Keywords

Health system resilience, organisational readiness for change, factor analysis, innovation adoption, evidence-based practice, task-sharing, primary care

Background

The treatment gap for mental disorders in South Africa, like other low- and middle-income countries (LMIC) and high-income countries (HIC), is well documented, with increasing attention being directed towards addressing this gap by providing mental health services in primary care. With the reality of constrained budgets for health and human resource limitations in South Africa and globally, task-sharing of interventions to address mental disorders is gaining traction, whereby

For peer review only

For peer review only - http://bmjopen.bmj.com/site/about/guidelines.xhtml

specific mental health services are delegated to non-specialist cadres with appropriate support and onward referral (1, 2). This approach is supported globally (3, 4) as a strategy for building health system resilience (5, 6) and is being investigated in a number of effectiveness and implementation studies in primary health care (PHC) settings in South Africa (2, 7-9). Little is known about the readiness and capability of many primary health facilities to implement this innovation, and there is great variation in implementation factors across facilities. Previous work by our group has identified patterns of factors associated with capability for implementing mental health counselling at primary care facilities in the Western Cape province. These included good patient information systems, organized services and a strong management team (all supporting the provision of patient-centred cared). (10).The variability across facilities observed in this work indicates the need for feasible ways to assess readiness and identify barriers to implementation for these service innovations.

As the introduction of task-shared interventions can be complex, some interventions fail to be implemented despite a supportive policy environment (11). There may be several reasons for implementation failure in this context (12). For example, psychosocial interventions can be complex in nature (e.g. multiple sessions, psychological content); community contexts where patients live may challenge participation in sessions (e.g. poverty, violence) (13, 14); and

For peer review only - http://bmjopen.bmj.com/site/about/guidelines.xhtml

For peer review only

importantly, the organisational setting of primary care facilities presents a complex environment for introducing mental health and other behavioural health innovations (e.g. heavy workloads, resource shortages, skills limitations, poor staff morale) (10, 15-21). Innovations need to be implemented by individuals embedded in complex organisations and difficult social contexts (22-24). This may help explain why many effective interventions do not transition to sustained routine practice in primary health organisations (25, 26), and why reducing the evidence-to-practice gap remains a priority for health services research.

Organisational readiness for change (ORC) provides a theoretical framework for conceptualising factors supporting or hindering initial adoption of new evidence-based practices. ORC, as defined by Weiner, is a shared psychological state that relies on organisation members' motivation to change (change commitment) and belief in their own capacity to change (change efficacy) (27). Commonly identified components contributing to this shared state include organisational dynamics, climate and culture (particularly leadership from management), and individual members' characteristics and readiness to accept change, including attitudes to evidence based practices (27-31). Assessing ORC enables implementers to identify and develop approaches to

For peer review only - http://bmjopen.bmj.com/site/about/guidelines.xhtml

reduce factors hindering innovation adoption and strengthen those factors that support adoption. In South Africa, constraints to innovation adoption include lack of resources, high workloads, inadequate staff skills, high levels of staff turnover, and complex interpersonal and hierarchical relationships (14, 19, 32, 33). The assessment of ORC in South African settings has substantial potential to enable identification and mitigation of these constraints.

A variety of instruments for assessing ORC have been developed (34) and used in HIC. Assessment of ORC in LMICs is limited to date but may have important potential for improving implementation of quality health care. The resources, operations, and outer system and inner organizational contexts and processes are likely to differ between LMICs and HICs. This study aimed to address this research gap through contextually adapting an instrument for assessing ORC in primary health settings in South Africa (35). The Texas Christian University ORC (TCU-ORC) scale (36) is among the most widely used of the ORC scales and has been tailored for use in health services research and with adaptation may be suitable for use in LMICs. Although there is good evidence for the instrument's validity in other settings (34), and it has been used in South Africa (37), little is known about the psychometric properties in LMICs and the South African context

For peer review only - http://bmjopen.bmj.com/site/about/guidelines.xhtml

in particular. The TCU-ORC is a comprehensive measure of ORC assessing both individual psychological and organizational and health system influences on ORC. However, with 125-items, its length may make it unpractical for routine use in busy and under-resourced services. There are continuing calls for development and testing of brief and pragmatic measures (e.g., low burden, important to stakeholders, actionable, psychometrically strong, related to theory) and such measures are more likely to be used in research and practice (38, 39).

This study was nested within a study known as Project MIND, a cluster randomised controlled trial evaluating two approaches to resourcing the integration of task-shared counselling for patients with depression and/or hazardous or harmful alcohol use (2). The Project MIND counselling programme comprises three-sessions of motivational interviewing and problem-solving therapy delivered by trained lay counsellors (2, 40). The integration of this service constitutes a change to the basket of health services currently available within primary care clinics, providing an opportunity for contextually validating the TCU-ORC. This study aimed to assess the psychometric properties of the TCU-ORC in South African primary care clinics and reduce the number of items to a smaller, more manageable and relevant tool for this setting.

For peer review only - http://bmjopen.bmj.com/site/about/guidelines.xhtml

Methods

Methods for the Project MIND trial (2) and this adaptation sub-study (35) are fully described elsewhere. Briefly, this study involved administering a set of ORC assessments to providers within Project MIND trial sites at two time points with the purpose of establishing the internal consistency, temporal stability and construct validity of the long-form TCU-ORC questionnaire.

Participants and Procedures

Data collection took place from July to December 2018 at the 24 facilities that participated in the MIND trial. Following a workshop that introduced the study in the facilities, health service employees (managers and staff) were requested to complete a paper-based, English language self-report survey, after granting written informed consent. English is the official language of business of the South African health system.

Participants at workshops included facility managers, operational managers, medical officers, psychiatric nurses, general nursing staff from the chronic disease care team, and lay counsellors. We aimed to secure participation of 12 staff members from each facility (288 participants in total), however the number of participants per workshop was at the discretion of the facility manager based on staff availability. Between one and two weeks after the workshop,

For peer review only - http://bmjopen.bmj.com/site/about/guidelines.xhtml

Project MIND fieldworkers asked a sub-sample of participants (convenience sample) from each facility to re-complete the paper-based survey comprising the ORC measures to enable assessment of test-retest reliability. These fieldworkers also approached nursing staff directly to fill in the questionnaire to gain enough responses for the first survey. In total, 395 surveys were completed: 281 participants completed the first survey and 118 participants completed both the first and test-retest survey.

Measures

The survey comprised three measures of ORC; the TCU-ORC (36), the Organisational Readiness to Change Assessment (ORCA) (41) and the Checklist for Assessing Readiness for Implementation (CARI) (42).

The four domains of the TCU-ORC (125 items total) measure Motivational Readiness for Change (Subscale A: covering staff's readiness, efficacy and support needs for implementing new practices; 33 items; e.g. clinical staff at your program needs guidance in matching client needs with services); Institutional Resources (Subscale B: adequacy of infrastructure, training and management; 31 items; e.g. much time and attention are given to staff supervision when needed); Staff Attributes (Subscale C: staff skills, management planning and leadership; 31 items; e.g.

For peer review only - http://bmjopen.bmj.com/site/about/guidelines.xhtml

learning and using new procedures are easy for you) and Organisational Climate (Subscale D: shared goals, communication structure, work environment; 30 items; e.g. the heavy staff workload reduces the effectiveness of your program). Items are scored on a five-point Likert scale ranging from strongly disagree to strongly agree. In development, these subscales showed acceptable reliability (alpha > .70 Subscale A; alpha > .60 Subscale B; alpha > .50 Subscale C; alpha > .50 Subscale D) (36).

The Organizational Readiness For Change Assessment (ORCA) (41) comprises 50 items across three scales. These assess (1) strength of evidence for the change to be introduced (9 items; e.g. the proposed change to the service delivery of the facility is supported by clinical experience with patients in other health care systems); (2) quality of the organisational context (22 items; e.g. in general in my facility, when there is agreement that change needs to happen we have the necessary support in terms of budget or financial resources) and (3) capacity for organisational facilitation of the change (19 items; e.g. communication will be maintained through regular meetings covering the intervention). Items are scored on a five-point Likert scale ranging from strongly disagree to strongly agree. There is evidence for the overall reliability and factor structure of the ORCA (alpha .74, .85 and .95 for the subscales 1, 2 and 3, respectively) (41).

For peer review only - http://bmjopen.bmj.com/site/about/guidelines.xhtml

The CARI checklist (42) includes 36 items that assess: (1) organisational capacity (five items; e.g. to what extent do you think technical assistance is available for the intervention being implemented); (2) organisational capacity (four items; e.g. to what extent do you think financial resources are adequate and available to introduce and sustain the intervention); (3) functional considerations (four items, e.g. to what extent do you think there is a system in place to share patient outcomes with staff and management), (4) organisational culture/climate (five items, e.g. to what extent do you think there are open lines of communication in place in this facility; (5) senior leadership (five items; e.g. to what extent do you think senior leadership is convinced of the value of this intervention; (6) staff capacity (five items; e.g. to what extent do you think staff show they are ready for this type of practice change); (7) implementation plan (three items; e.g. to what extent do you think there is intent to monitor fidelity of the intervention and (8) training; (five items, e.g. to what extent do you think adequate time will be set aside for training, reflection and practice). This instrument was selected because it contains items that are broadly similar in content and wording to the TCU-ORC items although data on its psychometric properties remain to be published. Neither of the additional instruments have been validated in LMIC to our knowledge.

Twenty-three items across the four subscales of the TCU-ORC were removed prior to the assessment as they were not appropriate for the South African PHC context, (See Supplementary

For peer review only - http://bmjopen.bmj.com/site/about/guidelines.xhtml

Table 1 for item removal based on discussion with the research group), leaving 104 items in the ORC for further assessment. Some of the removed items related to computer availability. While staff in primary care settings are meant to have access to computers and the internet, in practice this is often not the case with staff having limited access to computers where these are available. Minor editing of items was carried out to improve readability and appropriateness for clinic staff (for example 'your program' was edited to read 'your facility').

Analyses

Stata 16 statistical software was used for analysis. Cases with >50% missing data were removed (43). Missing data remaining in the data set was excluded from subsequent analysis using listwise exclusion. Normality tests were conducted for all measures using the Shapiro-Wilks test in combination with visual inspection of the normality plots. We used exploratory factor analysis, specifically Principal Axis Factoring (PAF) to identify latent dimensions represented in the variables (44, 45). The factor solution was rotated using Oblique Oblimin rotation, which is suited when factors are possibly related, but measure different aspects of a construct (46). Factor extraction was determined on the basis of the theoretical structure of the TCU-ORC (36), as well as the Kaiser K0 rule for common factors, in combination with Horn's parallel analysis (47-49).

For peer review only - http://bmjopen.bmj.com/site/about/guidelines.xhtml

Decisions around item retention were guided by item communalities and loading of items onto factors. Items with communalities of at least .70 are considered highly desirable (50). Although items with loadings greater than .40 are often retained (51), the decision was made to include items with factor loadings above .60 for parsimony due to the large number of items and to allow for a more stable factor solution.

Next, internal consistency of each subscale was assessed by calculating Cronbach's alpha of the reduced item subscales. Internal consistency is considered acceptable at values above .70 for generalisability (52). To assess temporal stability, we examined the extent to which the subscales and total scales for the first and retest surveys correlated with each other, with a correlation coefficient above .70 considered adequate. Convergent validity was assessed by calculating the correlation coefficient between the TCU-ORC, ORCA and CARI measures for the total scales (36, 41, 42).

Results

After removal of cases with less than 50% data, a total 275 participants with adequate responses who had completed the first survey and 118 participants who had completed both the first and retest survey were included in the analyses. Participants' sociodemographic characteristics are

For peer review only - http://bmjopen.bmj.com/site/about/guidelines.xhtml

summarized in Table 1. The sample comprised predominantly female nursing staff, reflecting the staffing profile of primary clinics in South Africa. A large proportion had worked at their current facility for more than five years and the majority had been in their profession for more than five years (Table 1).

Exploratory factor analysis

Assumption testing for factor analysis

Normality of the TCU-ORC was established by a non-significant Shapiro-Wilk result (p = .11) and visual inspection of the Histogram and the Q-Q plot. Bartlett's test of sphericity indicated that correlations between items were sufficiently large for factor analysis ($\chi^2(5356) = 12099.13$, $p < .01$). The Kaiser-Meyer-Olkin (KMO) measure of sampling adequacy was found to be acceptable with a value of .70 (53).

Factor extraction. The initial extraction was run with the Kaiser rule specified, retaining factors with eigenvalues greater than 0. This identified 74 factors. A parallel analysis (PA) was run in Stata, using Glorfeld's (1995) extension and using the 95th and 99th percentile to compare eigenvalues in order to increase sensitivity (49). The 95th percentile suggested retaining 14 factors, and the 99th percentile analysis resulted in 15 factors.

For peer review only - http://bmjopen.bmj.com/site/about/guidelines.xhtml

For peer review only

When conducting a PA, factors with a large number of variables are prone to inaccuracies due to poorly defined factors (54). As this measure contains a large number of variables, it is likely that the results of the PA over extracted factors. The decision was made to specify four factors, according to these results and the theoretical basis of the original TCU-ORC (36). After the initial extraction of four factors, we observed that multiple items from two subscales loaded onto one factor. Visual inspection of the scree plot confirmed three distinct points above the point of inflection (55), suggesting a three-factor solution. Subsequent analyses were based on the specification of a three-factor solution.

Factor loadings of the final factor structure are presented in Table 2. Eleven extractions were performed overall to reach the final three factor solution. This explained 72.4% of shared variance. We removed items with cross-loadings of .3 or above on any item and items were removed one at a time in sequential factor analysis runs (51). Sampling adequacy using the KMO Measure was assessed for each item as a post-estimation after each extraction. In each extraction, items with loadings less than .60 were removed after assessing how removing items affected the Cronbach's alpha for each subscale and whether internal consistency improved. The final three factor solution consists of 26 items, having removed 99 items and therefore providing a simplified, shortened version of the scale (Table 3).

For peer review only

For peer review only - http://bmjopen.bmj.com/site/about/guidelines.xhtml

Factor 1 'Clinic Organisational Climate' consists of 13 items, with loadings between .61 and .72. This factor is made up of items from the original Institutional Resources, Staff Attributes and Organizational Climate subscales. The revised subscale had a Cronbach's alpha of .90 and correlated with the original 'Organizational Climate' subscale (r = .82, p < .05).

Factor 2 'Motivational Readiness for Change' consists of eight items, with loadings between .64 and .78. The items in this subscale were all originally in the TCU-ORC's Motivational Readiness for Change subscale. This subscale had good reliability with a Cronbach's alpha of .93 and correlated with the original 'Motivational Readiness for Change' subscale (α = .79).

Factor 3 'Individual Change Efficacy' consists of five items, with factor loadings between .62 and .76. The items in this subscale all originated from the 'Staff Attributes' subscale. This subscale had a Cronbach's alpha of .83 and correlated with the original 'Individual Change Efficacy' subscale (α =.57).

Overall, the 26-item version of the TCU-ORC was strongly correlated with the original scale (r = .79, p < .05).

Temporal stability

For peer review only - http://bmjopen.bmj.com/site/about/guidelines.xhtml

For peer review only

A test-retest correlation coefficient between the initial and follow-up versions of the TCU-ORC was conducted, using only those items retained after PAF for both administration versions. Temporal stability was assessed for the overall scale as well as for each sub-scale. The correlation results are presented in Table 4. Subscales show adequate temporal stability for Clinic Organisational Climate (r = .87, $p < .01$), Motivational Readiness for Change (r = .60, $p < .01$), with exception of the Individual Change Efficacy subscale (r = .53, $p < .01$), Overall, the shortened scale was found to have good consistency across time with a test-retest correlation of r = .80, $p < .01$.

Convergent validity

The reliability coefficients of criterion measures were good for the ORCA (α = .96) and CARI (α = .96), supporting the interpretation of convergent correlations. The shortened version of the TCU-ORC was found to have acceptable convergent validity with the ORCA scale (r = .56, $p < .05$), with a moderate correlation with the CARI (r = .39, p < .05). Convergent validities of r = .70 are considered ideal, with validities less than r = .50 considered insufficient in organisational research (56) (54).

For peer review only - http://bmjopen.bmj.com/site/about/guidelines.xhtml

Discussion

Validated measures for assessing organizational functioning and dynamics in health organisations in LMICs are currently lacking (57). These measures are needed to assist health systems as they grapple with the need to be resilient and responsive to the changing health needs of the population (6, 32). To be applicable and useful, measures must be pragmatic and useful to the service providers involved in delivering and managing systems and facilities (38, 39). This study presents findings on the psychometric properties of the TCU-ORC amongst a sample of health workers from primary care settings in the Western Cape Province of South Africa. The sample was drawn from front-line providers in health facilities, who are key to influencing implementation of evidence-based practices through their role as 'street level bureaucrats' (58) who through their work have influence on the services received by facility clients. While the usefulness of this measure has been investigated by studies from a number of HIC (e.g. (59, 60)), this is the first investigation of its kind in primary care in South Africa and to our knowledge, in other LMICs.

The overall goal of this work was to develop a contextually relevant measure, feasible for use in primary care within South Africa (35). While the original measure has been widely used in better resourced settings, 125 items is not a feasible for use as a routine assessment in the busy and

For peer review only - http://bmjopen.bmj.com/site/about/guidelines.xhtml

under-resourced South African health system. A shorter, pragmatic instrument capable of collecting essential information to inform implementation is required for completion by primary care staff with high workloads and multiple competing priorities (14). The need for reducing response burden and respondent fatigue is aligned with current thinking among global mental health researchers who have noted response burden as a threat to the quality of data collected by lengthy measures (61). The current study resulted in the reduction of the TCU-ORC to 26 items. While this is a considerable reduction, further shortening may be needed to produce a scale truly feasible for routine use in primary care in other settings. Experience from HIC settings underscores the importance of ensuring implementation support measures are responsive to stakeholders' needs, present a low burden and have wide application across a multitude of settings (38, 39, 62). Scales with large number of items have been shown to be feasibly shortened to as low as 3 items per subscale retaining high degree of validity and reliability (alpha > .9) e.g. (63). With this in mind, we plan to conduct a Delphi study with stakeholders and experts from the South African primary care arena to further shorten the tool and enable integration of stakeholder perspectives. Subsequently a confirmatory factor analysis (CFA) will be conducted.

Factor analysis showed overlap between three of the original domains, suggesting a three-factor structure for the South African data, in contrast to the original four domains [(Motivational

For peer review only - http://bmjopen.bmj.com/site/about/guidelines.xhtml

Readiness for Change (A), Institutional Resources (B) Staff Attributes (C) and Organisational Climate (D)]. The proposed domains for the South African data, as shown in Table 2 are (1) Clinic Organisational Climate, (13 items) (2) Pressures for Change (8 items) and (3) Individual change efficacy (5 items). Each domain and the overall scale showed strong internal consistency, adequate temporal stability, and moderate convergent validity with the ORCA and CARI measures. The temporal stability for the Individual Change Efficacy scale may reflect the unstable and uncertain climate within which health providers often operate. At the time of this study, many of the clinics were affected by community violence and service delivery protests that sometimes led to clinic closures or restricted service delivery. It is plausible that these contextual factors may have impacted on individual providers perceptions of change efficacy, especially as efficacy for change is often context dependent. However, more research is needed to identify and address such "bridging factors" that demonstrate bi-directional influences of outer system and inner organizational contexts (64).

Three items with cross loadings >.3 (.3 to .5) were removed suggesting retained items were representing relatively unique constructs that were understood by respondents (57). The domain 'Clinic Organisational Climate' incorporates items from the Institutional Resources, Staff Attributes and Organisational Climates domains of the original TCU-ORC. This may be because

For peer review only - http://bmjopen.bmj.com/site/about/guidelines.xhtml

institutional resources, staff attributes, and trusting supervisory relationships become essential prerequisites for staff's functioning within constrained environments. These are perceived as key aspects of the organizational climate and have been previously shown to group as elements of organisational functioning (10). Thirteen items were retained in this domain suggesting these are key to assessing organizational climate in this setting. Items included those relating to management, supervision, teamwork, leadership and trusting relationships, which aligns with other work investigating primary care functioning in South Africa (19, 32, 65, 66). Findings from qualitative work assessing primary care facilities' preparedness for mental health counselling implementation also suggest intersecting relationships between resource availability, management style and facility environment (21). The Pressures for Change domain remained largely consistent with the corresponding domain of the original TCU-ORC. Only a small number of items were retained from the C domain on Staff Attributes - these related to individual agency for change. Organisational theory outlines how individuals within an organisation can be resistant to change, particularly in the context of heavy workloads (27, 67) known to be present in South African facilities. South African facility managers are often clinicians, without management training, who work in high workload environments and indicate that workload constrains innovation adoption (68). The South African health system has a strong hierarchy of downward supervision and

For peer review only - http://bmjopen.bmj.com/site/about/guidelines.xhtml

management, compliance and targets and this may hinder frontline managers and staff from being responsive to patient needs and public health burden (69). At the same time, nurses are the majority of clinic staff and are key to organisational climate and change. The items retained in this scale may therefore be most relevant for assessing individual perceptions amongst nursing staff on their agency and willingness to change.

In relation to convergent validity, correlation of TCU-ORC-SF with the ORCA showed large effect, but with the CARI correlation showed medium effect. These two additional instruments were also designed to assess organisational readiness for change in HICs, and although some aspects of organisational culture, dynamics and functioning are translatable to other settings, others may not be robustly relevant in the South African PHC context. With regards to correlation of the shortened tool with the CARI, it is likely that the restricted range of responses in the CARI resulted in a lower correlation (70). The CARI items are measured on a 4-point scale, while the TCU-ORC items are measured on a 5-point scale. Nonetheless, it is the opinion of the researcher/s that these validity correlations between the ORCA, CARI and TCU-ORC scales are acceptably large to assume convergence. We propose that the revised domains presented here more closely capture the essentials of organisational readiness for change in South African PHC settings than the original measures. This adapted measure may enable progress on assessing readiness for

For peer review only - http://bmjopen.bmj.com/site/about/guidelines.xhtml

implementation of evidence-based practices and addressing barriers in South African health facilities, identified as a prerequisite for implementation in our previous work (10). Further, there will be a wide variety of factors influencing readiness for change, particularly in different settings in LMIC and elsewhere, and detailed implementation research elucidating these elements would support the use or adaptation of this tool in different settings. The current work provides a base for driving policy implementation to enable adoption of innovations to improve public health through strengthening primary healthcare platforms.

Limitations

Data for this study were collected from primary care facilities from the Western Cape province. This province is more well-resourced than other provinces. Data from the study showed a sociodemographic profile of facility staff that reflects the qualifications, professions, and work experience common across primary care settings in the country. This suggests appropriateness for the broader South African context, although there are important disparities in human and other resources and variations in burden of disease and contexts across provinces (5, 71) and additional adaptations could be required in other areasThe survey was in English which is not the first language of health providers in some settings, however English is the official business language

For peer review only - http://bmjopen.bmj.com/site/about/guidelines.xhtml

of government agencies and health providers would be expected to be proficient. The ORCA and CARI measures have also not been adapted for use in LMIC use so there may be limitations in their validity for this context which may have influenced convergent validity findings.

Conclusion

This study has presented the contextual adaptation of the TCU-ORC in a LMIC setting. Based on the findings from this study, we propose a short form of the TCU-ORC, comprising 26 of 125 items. The three subscales proposed: Clinic Organisational Climate, Motivational Readiness for Change, and Individual Change Efficacy contain key items for assessing ORC in this context. This scale may be useful as a tool for health service managers to highlight potential roadblocks to the introduction of innovations that may need to be addressed when developing an implementation strategy. This shortened tool however may still be too lengthy for widespread practical use in primary care services and further shortening involving stakeholders who will use the tool in practice is required.

Declarations

Ethics approval and consent to participate

For peer review only - http://bmjopen.bmj.com/site/about/guidelines.xhtml

406 Ethical approval was granted for this study by the Human Research Ethics Committee of the South African Medical Research Council (EC004-2-2015) and approval for working in facilities was also granted by the Western Cape Provincial Department of Health.

409 **Consent for publication**

410 Not applicable.

411 **Patient and public involvement**

412 It was not appropriate to involve patients or the public in this study since it focused on primary health staff as employees of the health system. We will present the shortened tool to staff and managers of the study facilities as part of the post-trial engagement meetings for Project MIND.

415 **Availability of data and materials**

416 Extra data can be accessed via the Dryad data repository at http://datadryad.org/ with the doi.org/10.5061/dryad.w6m905qqm.

418 **Competing interests**

419 The authors declare that they have no competing interests.

For peer review only

For peer review only - http://bmjopen.bmj.com/site/about/guidelines.xhtml

Funding statement

This study is funded jointly by the British Medical Research Council, Wellcome Trust, Department for International Development, the Economic and Social Research Council, the Global Challenges Research Fund (MR/M014290/1) as well as funding from the South African Medical Research Council (SAMRC, contract). CBS was supported as a postdoctoral fellow by funding from the SAMRC through its Division of Research Capacity Development under the Intra-Mural Postdoctoral Fellowship Programme from funding received from the South African National Treasury. The content hereof is the sole responsibility of the authors and does not necessarily represent the official views of the SAMRC or the funders.

Author contributions

CBS and BM conceptualised the study. CBS and PPW managed data collection. BM and KS are Principal Investigators on the Project MIND trial in which the study is nested. CBS and EW conducted factor analysis. EW provided statistical technical input and drafted some sections of the results. GA provided technical guidance on methods and analysis. All authors reviewed and contributed to the final version of the manuscript.

Acknowledgements

For peer review only - http://bmjopen.bmj.com/site/about/guidelines.xhtml

The authors thank Lesley-Anne Erasmus-Claasen, Yuche Jacobs and Nombuso Moshiga for their fieldwork on the study as well as all participating facilities, managers and staff.

For peer review only

440 **Table 1 Sociodemographic data from PHC facility-based staff**

Item	Category	Frequency	Percentage
Gender	Female	214	88.4%
	Male	28	11.6%
Age	20-30	14	5.3%
	31-40	72	27.2%
	41-50	73	27.5%
	51-60	84	31.7%
	61-65	22	8.3%
Ethnicity	Black African	136	50.2%
	Coloured	117	43.2%
	White	13	4.8%
	Indian/Asian	3	1.1%
	Other	2	0.7%
Time at facility	0-6 months	22	8.1%
	6-11 months	20	7.4%
	1-3 years	61	22.5%
	3-5 years	48	17.7%
	More than 5 years	120	44.3%
Education level	Completed high school	62	23.0%
	Some tertiary education	65	24.1%
	Bachelor's degree	51	18.9%
	Master's degree	7	2.6%
	Doctoral degree	3	1.1%
	Diploma in healthcare	74	27.3%
	Other	8	3.0%
Profession*	Medicine	25	9.1%
	Social work	6	2.1%
	Counselling	62	22.7%
	Psychology	2	0.7%
	Nursing	149	54.8%
	Administration	30	11.0%

For peer review only

For peer review only - http://bmjopen.bmj.com/site/about/guidelines.xhtml

	Other	18	6.6%
Time in profession	0-6 months	7	2.6%
	6-11 months	7	2.6%
	1-3 years	36	13.4%
	3-5 years	25	9.4%
	More than 5 years	192	72.0%

*Some participants selected working in more than one profession

For peer review only

Table 2 Principal axis factoring by domains of the original TCU-ORC

For peer review only - http://bmjopen.bmj.com/site/about/guidelines.xhtml

Subscale A: Motivational Readiness for Change (29 items included in factor analysis)

Subscale B: Institutional Resources (22 items included in factor analysis)

Subscale C: Staff Attributes (24 items included in factor analysis)

Subscale D: Organisational Climate (29 items included in factor analysis)

Initial Factor 1		Final factor 1 Clinic Organisational Climate		Initial Factor 2		Final factor 2 Motivational Readiness for Change		Initial Factor 3		Final Factor 3 Individual Change Efficacy		Initial Factor 4	
Items	Loadings	Items	Loadings	Items	Loadings	Items	Loadings	Items	Loadings	Items	Loadings	Items	Loadings
A26	.32	B1	.66	A1	.56	A1	.68	B20	.35	C8	.68	B2	.38
B1	.64	B3	.61	A2	.51	A3	.77	C1	.44	C9	.71	C24	.41
B3	.53	B10	.68	A3	.55	A4	.78	C2	.30	C10	.76	D5	.36
B4	.39	B16	.71	A4	.59	A5	.77	C4	.48	C11	.62	D12	.35
B5	.56	B19	.65	A5	.63	A6	.73	C8	.50	C18	.62	D15	.34
B6	.51	C7	.66	A6	.59	A7	.70	C9	.61			D16	.34
B7	.60	D7	.62	A7	.57	A8	.70	C10	.52			D19	.44
B8	.59	D11	.65	A8	.58	A10	.64	C11	.47			D22	.54
B10	.69	D14	.66	A9	.61			C12	.34			D27	.33
B11	.64	D21	.70	A10	.69			C13	.31				
B12	.39	D25	.72	A11	.63			C14	.35				
B13	.44	D26	.66	A12	.64			C17	.44				
B14	.48	D28	.64	A13	.56			C18	.53				
B15	.39	D29	.61	A14	.55			C23	.45				
B16	.70			A15	.60			D6	.33				
B17	.58			A16	.59								
B18	.52			A18	.30								
B19	.62			A19	.50								
B21	.45			A20	.46								
C3	.44			A21	.49								
C5	.34			A22	.37								
C6	.53			A23	.51								
C7	.65			A24	.34								
C10	.33			A28	.38								
C13	.32			A29	.30								
C14	.49			C2	.31								
C15	.35			C8	.36								

For peer review only - http://bmjopen.bmj.com/site/about/guidelines.xhtml

C20	.38	C10	.34
C21	.41	C16	.40
C23	.39	C17	.32
D3	.42	C18	.43
D4	.53	D6	.45
D5	.62	D15	.32
D7	.61	D24	.36
D8	.67		
D9	.64		
D11	.60		
D13	.56		
D14	.67		
D16	.40		
D17	.50		
D18	.55		
D20	.45		
D21	.67		
D23	.45		
D25	.70		
D26	.63		
D28	.61		
D29	.61		

Extraction Method: Factor Analysis.
Rotation Method: Oblique Oblimin

For peer review only

Table 3 Excluded items and proposed revised domains*

Original TCU ORC domain	No. of items in initial factor analysis (104 total)	Excluded items<br><br>(loadings < .6 through 11 extractions)	Proposed revised domains	Number of items in proposed scale
A Motivational Readiness for Change	29	Your facility needs guidance in<br>A2 using patient assessments to guide clinical care<br>A9 defining its mission<br>A11 assigning or clarifying staff roles<br>A12 setting out accurate job descriptions for staff<br>A13 evaluating staff performance<br>A14 improving relations among staff<br>A15 improving communications among staff<br>A16 improving record keeping and information systems<br>A17 basic computer skills/programs<br>A18 specialized computer applications<br>A19 new developments and practices in your area of responsibility<br>A20 new procedures being used or planned<br>A21 continuous professional development<br>A22 new laws or regulations you need to know about<br>A23 management or supervisory responsibilities<br><br>Current pressures to make facility changes come from<br>A24 community members (patients)<br>A25 other staff members<br>A26 facility supervisors or managers<br>A27 district level managers<br>A28 community groups<br>A29 national and provincial DOH structures	**Motivational Readiness for Change**<br>Clinical staff at your facility needs guidance in:<br>A1 assessing patient needs<br>A3 using patient assessments to document patient improvements<br>A4 matching patient needs with services<br>A5 improving relationship with patients<br>A6 improving patient thinking and problem-solving skills to manage chronic disease<br>A7 improving behavioural management of patients<br>A8 identifying and using evidence-based practices<br>Your facility needs guidance in:<br>A10 setting specific goals for improving services	8
B Institutional Resources	22	B2 Frequent staff turnover is a problem for your facility<br>B4 The space in your facilities are adequate for conducting group or individual counselling<br>B5 You have clinical supervisors who are capable and qualified<br>B6 You learned new skills or techniques at a professional training in the past year<br>B7 Much time and attention are given to staff supervision when needed<br>B8 Staff in your facility are able to spend the time needed with patients<br>B9 Equipment at your facility is mostly old and outdated<br>B11 Support staff in your facility have the skills they need to do their jobs<br>B12 Offices in your facility allow the privacy needed for individual counselling<br>B13 Your facility holds regular in-service training<br>B14 Your facility has enough staff to meet current patient needs<br>B15 Clinical staff in your program are well-trained<br>B17 Offices and equipment in your facility are adequate<br>B18 Your facility provides a comfortable reception/waiting area for patients<br>B20 A larger support staff is needed to help meet needs at your program.<br>B21 Staff in your facility are able to attend professional training<br>B22 Staff concerns are ignored in most decisions made in your facility<br>D29 You feel encouraged to try new and different techniques	**Clinic organisational climate**<br>B1 You have good facility management at your facility<br>B3 Staff training and continuing education are priorities in your facility<br>B10 Clinical and management decisions for your facility are well planned<br>B16 You have confidence in how decisions at your facility are made<br>B19 Staff meet frequently with clinical supervisors about patient needs and progress<br>C7 Your facility encourages and supports professional growth<br>D7 Ideas and suggestions in your facility get fair consideration by management<br>D11 Your facility staff is always kept well informed<br>D14 Your facility operates with clear goals and objectives<br>D21 The formal and informal communication channels in your facility work very well<br>D25 Management fully trusts professional judgments of staff in your facility<br>D26 Staff members always feel free to ask questions and express concerns in your facility<br>D28 Management for your facility has a clear plan for its future	13
C Staff Attributes	24	C1 You have the skills needed to effectively perform your role<br>C2 Other staff often ask your advice	**Individual change efficacy**<br>C8 You are effective and confident in doing your job	5

For peer review only - http://bmjopen.bmj.com/site/about/guidelines.xhtml

		C3 You are satisfied with your present job<br>C4 Learning and using new procedures are easy for you<br>C5 You are considered an experienced source of advice about services and/or facility management<br>C6 You feel appreciated for the job you do at work<br>C12 You regularly influence the decisions of other staff you work with<br>C13 You usually accomplish whatever you set your mind on<br>C14 You do a good job of regularly updating and improving your skills<br>C15 You regularly read professional articles or books related to your work<br>C16 Other staff often ask for your opinions about issues in the facility<br>C17 You are willing to try new ideas even if some staff members are reluctant<br>C19 You are sometimes too cautious or slow to make changes<br>C20 You are proud to tell others where you work<br>C21 You like the people you work with<br>C22 You are viewed as a leader by the staff you work with<br>C23 You consistently plan ahead and carry out your plans<br>C24 You would like to find a job somewhere else	C9 You are able to adapt quickly when you have to make changes<br>C10 Keeping your professional skills up-to-date is a priority for you<br>C11 You give high value to the work you do<br>C18 You frequently share your knowledge of new ideas with others	
D Organisational Climate	29	D1 Some staff members seem confused about the main goals for your facility<br>D2 The heavy staff workload reduces the effectiveness of your facility<br>D3 You frequently hear good ideas from other staff for improving services<br>D4 Care plans and decisions for patients in your facility often get revised by a clinical supervisor<br>D5 The general attitude in your facility is to accept new and changing technology or procedures<br>D6 More open discussions about facility issues are needed where you work<br>D8 Staff members at your facility work together as a team<br>D9 Your duties are clearly related to the goals for your facility<br>D10 You are under too many pressures to do your job effectively<br>D12 New ways of working from staff are discouraged where you work<br>D13 Mutual trust and cooperation among staff in your program are strong<br>D15 Staff members at your facility often show signs of high stress and strain<br>D16 It is easy to change procedures at your facility to meet new conditions<br>D17 Staff in your facility can try out different techniques to improve their effectiveness<br>D18 Staff members at your facility get along very well<br>D19 Staff members are given too many rules in your facility<br>D20 Staff members at your program are quick to help one another when needed<br>D22 There is too much friction among staff members you work with<br>D23 Staff members at your facility understand the work of the facility fits as part of the health improvement in your community<br>D24 Some staff in your facility do not do their fair share of work<br>D27 Staff frustration is common where you work		

* 'A' items refer to the Motivational Readiness for Change scale, 'B' items refer to the Institutional Resources scale, 'C' items refer to the Staff Attributes scale, 'D' items refer to the Organisational Climate scale.

For peer review only

For peer review only - http://bmjopen.bmj.com/site/about/guidelines.xhtml

Table 4 Internal consistency and retest reliability of the new subscales

Subscale/ Factor	Description	Cronbach's Alpha	Temporal stability (*r*)
Clinic Organizational Climate	Management practices, trust, leadership, communication, appreciation of staff, facility resources, attitudes to change	.90	.87**
Motivational Readiness for Change	Areas of need for facility improvement, and staff development	.91	.60**
Individual Change Efficacy	Individual self-efficacy, adaptability, knowledge and skills	.83	.53**
Total revised scale		.86	.80**

** Significant at the .01 level

References

1. Department of Health RoSA. National Mental Health Policy Framework and Strategic Plan 2013-2020. Pretoria: Department of Health South Africa, 2013.
2. Myers B, Lund C, Lombard C, Joska J, Levitt N, Butler C, et al. Comparing dedicated and designated models of integrating mental health into chronic disease care: study protocol for a cluster randomized controlled trial. Trials. 2018;19(1):185.
3. De Silva MJ, Ryan G. Global mental health in 2015: 95% implementation. The lancet Psychiatry. 2016;3(1):15-7.
4. Ciapponi A, Lewin S, Herrera CA, Opiyo N, Pantoja T, Paulsen E, et al. Delivery arrangements for health systems in low-income countries: an overview of systematic reviews. Cochrane Database Syst Rev. 2017;9:Cd011083.
5. Fusheini A, Eyles J. Achieving universal health coverage in South Africa through a district health system approach: conflicting ideologies of health care provision. BMC health services research. 2016;16(1):558-.
6. Barasa EW, Cloete K, Gilson L. From bouncing back, to nurturing emergence: reframing the concept of resilience in health systems strengthening. Health policy and planning. 2017;32(suppl_3):iii91-iii4.
7. Lund C, Alem A, Schneider M, Hanlon C, Ahrens J, Bandawe C, et al. Generating evidence to narrow the treatment gap for mental disorders in sub-Saharan Africa: rationale, overview and methods of AFFIRM. Epidemiol Psychiatr Sci. 2015;24(3):233-40.

For peer review only - http://bmjopen.bmj.com/site/about/guidelines.xhtml

8. Fairall L, Petersen I, Zani B, Folb N, Georgeu-Pepper D, Selohilwe O, et al. Collaborative care for the detection and management of depression among adults receiving antiretroviral therapy in South Africa: study protocol for the CobALT randomised controlled trial. Trials. 2018;19(1):193.
9. Petersen I, Bhana A, Fairall LR, Selohilwe O, Kathree T, Baron EC, et al. Evaluation of a collaborative care model for integrated primary care of common mental disorders comorbid with chronic conditions in South Africa. BMC psychiatry. 2019;19(1):107.
10. Myers B, Breuer E, Lund C, Petersen Williams P, van der Westhuizen C, Brooke-Sumner C, et al. Assessing capability for implementing mental health counselling within primary care facilities in a middle-income country: A feasibility study. Journal of psychiatric and mental health nursing. 2019;26(5-6):163-74.
11. Hanson K, Cleary S, Schneider H, Tantivess S, Gilson L. Scaling up health policies and services in low- and middle-income settings. BMC health services research. 2010;10 Suppl 1:I1.
12. Jacobs SR, Weiner BJ, Reeve BB, Hofmann DA, Christian M, Weinberger M. Determining the predictors of innovation implementation in healthcare: a quantitative analysis of implementation effectiveness. BMC health services research. 2015;15:6.
13. Eyles J, Harris B, Fried J, Govender V, Munyewende P. Endurance, resistance and resilience in the South African health care system: case studies to demonstrate mechanisms of coping within a constrained system. BMC health services research. 2015;15(1):432.
14. Brooke-Sumner C, Petersen-Williams P, Kruger J, Mahomed H, Myers B. 'Doing more with less': a qualitative investigation of perceptions of South African health service managers on implementation of health innovations. Health policy and planning. 2019;34(2):132-40.
15. Lehmann U, Gilson L. Actor interfaces and practices of power in a community health worker programme: a South African study of unintended policy outcomes. Health policy and planning. 2013;28(4):358-66.
16. Gopinathan U, Lewin S, Glenton C. Implementing large-scale programmes to optimise the health workforce in low- and middle-income settings: a multicountry case study synthesis. Tropical medicine & international health : TM & IH. 2014;19(12):1437-56.
17. Innis J, Dryden-Palmer K, Perreira T, Berta W. How do health care organizations take on best practices? A scoping literature review. Int J Evid Based Healthc. 2015;13(4):254-72.
18. Freeman M. Global mental health in low and middle income, especially African countries. Epidemiol Psychiatr Sci. 2016;25(6):503-5.
19. Gilson L, Barasa E, Nxumalo N, Cleary S, Goudge J, Molyneux S, et al. Everyday resilience in district health systems: emerging insights from the front lines in Kenya and South Africa. BMJ global health. 2017;2(2):e000224.
20. Mbau R, Gilson L. Influence of organisational culture on the implementation of health sector reforms in low- and middle-income countries: a qualitative interpretive review. Global health action. 2018;11(1):1462579.
21. Oboirien K, Harris B, Goudge J, Eyles J. Implementation of district-based clinical specialist teams in South Africa: Analysing a new role in a transforming system. BMC health services research. 2018;18(1):600.
22. Lund C, Brooke-Sumner C, Baingana F, Baron EC, Breuer E, Chandra P, et al. Social determinants of mental disorders and the Sustainable Development Goals: a systematic review of reviews. The Lancet Psychiatry.5(4):357-69.
23. Aarons GA, Hurlburt M, Horwitz SM. Advancing a conceptual model of evidence-based practice implementation in public service sectors. Adm Policy Ment Hlth. 2011;38.
24. Cresswell K, Sheikh A. Organizational issues in the implementation and adoption of health information technology innovations: an interpretative review. Int J Med Inform. 2013;82(5):e73-86.

For peer review only - http://bmjopen.bmj.com/site/about/guidelines.xhtml

25. Lau R, Stevenson F, Ong BN, Dziedzic K, Treweek S, Eldridge S, et al. Achieving change in primary care--effectiveness of strategies for improving implementation of complex interventions: systematic review of reviews. BMJ open. 2015;5(12):e009993.
26. Lau R, Stevenson F, Ong BN, Dziedzic K, Treweek S, Eldridge S, et al. Achieving change in primary care--causes of the evidence to practice gap: systematic reviews of reviews. Implementation Science. 2016;11:1-39.
27. Weiner BJ. A theory of organizational readiness for change. Implementation Science: IS. 2009;4:67-.
28. Aarons GA. Measuring provider attitudes toward evidence-based practice: consideration of organizational context and individual differences. Child and adolescent psychiatric clinics of North America. 2005;14(2):255-71, viii.
29. Aarons GA, Cafri G, Lugo L, Sawitzky A. Expanding the domains of attitudes towards evidence-based practice: the evidence based practice attitude scale-50. Adm Policy Ment Health. 2012;39(5):331-40.
30. Attieh R, Gagnon MP, Estabrooks CA, Legare F, Ouimet M, Roch G, et al. Organizational readiness for knowledge translation in chronic care: a review of theoretical components. Implementation science : IS. 2013;8:138.
31. Williams B, Perillo S, Brown T. What are the factors of organisational culture in health care settings that act as barriers to the implementation of evidence-based practice? A scoping review. Nurse education today. 2015;35(2):e34-41.
32. Gilson L. Trust and the development of health care as a social institution. Soc Sci Med. 2003;56(7):1453-68.
33. Gilson L, Palmer N, Schneider H. Trust and health worker performance: exploring a conceptual framework using South African evidence. Social Science & Medicine (1982). 2005;61(7):1418-29.
34. Gagnon M-P, Attieh R, Ghandour EK, Légaré F, Ouimet M, Estabrooks CA, et al. A Systematic Review of Instruments to Assess Organizational Readiness for Knowledge Translation in Health Care. PloS one. 2014;9(12):e114338.
35. Brooke-Sumner C, Sorsdahl K, Lombard C, Petersen-Williams P, Myers B. Protocol for development and validation of a context-appropriate tool for assessing organisational readiness for change in primary health clinics in South Africa. BMJ open. 2018;8(4).
36. Lehman WE, Greener JM, Simpson DD. Assessing organizational readiness for change. Journal of substance abuse treatment. 2002;22(4):197-209.
37. Bowles S, Louw J, Myers B. Perceptions of organizational functioning in substance abuse treatment facilities in South Africa. International journal of mental health and addiction. 2011;9(3):308-19.
38. Glasgow RE, Riley WT. Pragmatic measures: what they are and why we need them. Am J Prev Med. 2013;45(2):237-43.
39. Glasgow RE. What does it mean to be pragmatic? Pragmatic methods, measures, and models to facilitate research translation. Health Educ Behav. 2013;40(3):257-65.
40. Sorsdahl K, Naledi T, Lund C, Levitt NS, Joska JA, Stein DJ, et al. Integration of mental health counselling into chronic disease services at the primary health care level: Formative research on dedicated versus designated strategies in the Western Cape, South Africa. 2020:1355819620954232.
41. Helfrich CD, Li YF, Sharp ND, Sales AE. Organizational readiness to change assessment (ORCA): development of an instrument based on the Promoting Action on Research in Health Services (PARIHS) framework. Implementation science : IS. 2009;4:38.
42. Barwick M. Checklist to assess organizational readines s (CARI) for EIP Implementation. Toronto, Ontario: Hospital for Sick Children: 2011.

For peer review only - http://bmjopen.bmj.com/site/about/guidelines.xhtml

43. Van den Broeck J, Argeseanu Cunningham S, Eeckels R, Herbst K. Data Cleaning: Detecting, Diagnosing, and Editing Data Abnormalities. PLOS Medicine. 2005;2(10):e267.
44. Henson R, Roberts J. Use of Exploratory Factor Analysis in Published ResearchCommon Errors and Some Comment on Improved Practice. Educational and Psychological Measurement - EDUC PSYCHOL MEAS. 2006;66:393-416.
45. Mooi E, Sarstedt, M., & Mooi-Reci, I. . Principal component and factor analysis. Market Research. Singapore: Springer; 2018. p. pp 265-311.
46. Fabrigar LR, Wegener DT, MacCallum RC, Strahan EJ. Evaluating the use of exploratory factor analysis in psychological research. Psychological Methods. 1999;4(3):272-99.
47. Ledesma RD, Valero-Mora, P. Determining the number of factors to retain in EFA: An easy-to-use computer program for carrying out parallel analysis. Practical assessment, research, and evaluation. 2007;12(1):2.
48. Hayton JC, Allen DG, Scarpello V. Factor Retention Decisions in Exploratory Factor Analysis: A Tutorial on Parallel Analysis. Organizational Research Methods. 2004;7(2):191-205.
49. Dinno A. Implementing Horn's Parallel Analysis for Principal Component Analysis and Factor Analysis. Stata Journal. 2009;9:291-8.
50. Field A. Disscovering statistics using IMB SPSS statistics: Thousand Oaks: SAGE Publications Inc; 2013.
51. Costello AB, Osborne J. Best Practices in Exploratory Factor Analysis: Four Recommendations for Getting the Most From Your Analysis. Practical Assessment, Research & Evaluation. 2005;10:1-9.
52. Bartholomew DJ, Knott, M., & Moustaki, I. Latent variable models and factor analysis: A unified approach: John Wiley & Sons.; 2011.
53. Pett MA, Lackey, N. R., & Sullivan, J. J. Making sense of factor analysis. Thousand Oaks: Sage Publications; 2003.
54. Jones JM. The Influence of a Proposed Margin Criterion on the Accuracy of Parallel Analysis in Conditions Engendering Underextraction Masters Theses & Specialist Projects: https://digitalcommons.wku.edu/theses/2446; 2018.
55. Cattell RB. The Scree Test For The Number Of Factors. Multivariate Behavioral Research. 1966;1(2):245-76.
56. Carlson KD, Herdman, A. O. Understanding the impact of convergent validity on research results. Organizational Research Methods. 2012;15(1):17-32.
57. Uy J, Macarayan EK, Ratcliffe HL, Miller K, Otupiri E, Awoonor-Williams JK, et al. Preliminary validation of the PRImary care facility Management Evaluation tool (PRIME-Tool), a national facility management survey implemented in Ghana. BMC health services research. 2019;19(1):937.
58. Walker L, Gilson L. 'We are bitter but we are satisfied': nurses as street-level bureaucrats in South Africa. Soc Sci Med. 2004;59(6):1251-61.
59. Rampazzo L, De Angeli M, Serpelloni G, Simpson DD, Flynn PM. Italian Survey of Organizational Functioning and Readiness for Change: A Cross-Cultural Transfer of Treatment Assessment Strategies. 12: Karger AG. p. 176-81.
60. Rowan-Szal GA, Greener JM, Joe GW, Simpson DD. Assessing program needs and planning change. Journal of substance abuse treatment. 2007;33(2):121-9.
61. Haroz EE, Kane JC, Nguyen AJ, Bass JK, Murray LK, Bolton P. When less is more: reducing redundancy in mental health and psychosocial instruments using Item Response Theory. Global Mental Health. 2020;7:e3.
62. Sousa VD, Rojjanasrirat W. Translation, adaptation and validation of instruments or scales for use in cross-cultural health care research: a clear and user-friendly guideline. J Eval Clin Pract. 2011;17(2):268-74.

For peer review only - http://bmjopen.bmj.com/site/about/guidelines.xhtml

63. Wells SY, Morland LA, Torres EM, Kloezeman K, Mackintosh MA, Aarons GA. The Development of a Brief Version of the Posttraumatic Cognitions Inventory (PTCI-9). Assessment. 2019;26(2):193-208.
64. Moullin JC, Dickson KS, Stadnick NA, Rabin B, Aarons GA. Systematic review of the Exploration, Preparation, Implementation, Sustainment (EPIS) framework. 2019;14(1):1.
65. Gilson L. Editorial: building trust and value in health systems in low- and middle-income countries. Soc Sci Med. 2005;61(7):1381-4.
66. Gilson L, Elloker S, Olckers P, Lehmann U. Advancing the application of systems thinking in health: South African examples of a leadership of sensemaking for primary health care. Health research policy and systems / BioMed Central. 2014;12:30.
67. Kelly P, Hegarty J, Barry J, Dyer KR, Horgan A. A systematic review of the relationship between staff perceptions of organizational readiness to change and the process of innovation adoption in substance misuse treatment programs. J Subst Abuse Treat. 2017;80:6-25.
68. Brooke-Sumner C, Petersen-Williams P, Kruger J, Mahomed H, Myers B. 'Doing more with less': a qualitative investigation of perceptions of South African health service managers on implementation of health innovations. Health policy and planning. 2019;34(2):132-40.
69. Cleary SM, Molyneux S, Gilson L. Resources, attitudes and culture: an understanding of the factors that influence the functioning of accountability mechanisms in primary health care settings. BMC health services research. 2013;13:320-.
70. Furr RM, Bacharach, V. R. Psychometrics: An Introduction. Sage; 2006. p. 191-235.
71. Maphumulo WT, Bhengu BR. Challenges of quality improvement in the healthcare of South Africa post-apartheid: A critical review. Curationis. 2019;42(1):e1-e9.

For peer review only

For peer review only - http://bmjopen.bmj.com/site/about/guidelines.xhtml

Supplementary Table 1: Items removed from TCU-ORC before survey use based on research team discussion (23 total)

Item removed from original scale	Reason
A5 increasing program participation by clients	With high workloads and staff shortages this is not part of the expected role
A9 improving cognitive focus of clients during group counseling	Group counselling not currently provided
A19 improving billing/financial/accounting procedures	Clinic services provided free of charge
A30 Current pressures to make program changes come from board members or overseers	Stakeholder group not influential in government run and funded health facilities
A31 Current pressures to make program changes come from community groups	Stakeholder group not influential in government run and funded health facilities
A32 Current pressures to make program changes come from funding agencies	Stakeholder group not influential in government run and funded health facilities
A33 Current pressures to make program changes come from accreditation or licensing authorities	Stakeholder group not influential in government run and funded health facilities
B6 Policies limit use of the Internet for work-related needs at your program.	Most clinic staff do not have internet access at work or use for work purposes
B7 Computer problems are usually repaired promptly at your program	Most clinic staff do not use computers in day to day work
B10 You have convenient access to e-mail at work	Most clinic staff to not use email for work purposes
B14 More computers are needed for staff in your program to use	Clinic staff not expected to have computers for work use
B15 Most client records for your program are computerized	Electronic records system not implemented
B21 You used the Internet at work recently to access drug treatment information.	Clinic staff do not use internet for work purposes
B23 You have easy access for using the Internet at work	Clinic staff not expected to have internet access for work purposes
B26 You have a computer to use in your personal office space at work	Clinic staff not expected to have computer for work purposes
B29 The budget in your program allows staff to attend professional training.	General staff do not have knowledge about budgets and allocations, in service training most common
B30 Staff in your program feel comfortable using computers	Clinic staff not expected to use computers for work purposes
C12 12-step programs (AA/NA) are recommended to many of your clients	12 step programmes not accessible in most settings
C21 Behavior modification (contingency management) is used with many of your clients	Behaviour modification not implemented – no substance abuse behaviour change in PHC
C22 You have the skills needed to conduct effective individual counseling.	General staff not expected to provide individual counselling, individual counselling not widely implemented
C26 Cognitive theory (RET, RBT) guides much of your counseling.	Counselling not implemented
C31 Pharmacotherapy and related medications are important for many of your clients	Staff see not only substance abuse or mental health clients – medication will be widely used for other conditions
D11 Counselors in your program are given broad authority in treating their clients	Counselling not implemented by nursing staff

For peer review only
